# *δ* and *φ* back-donation in An^IV^ metallacycles

**DOI:** 10.1038/s41467-020-15197-w

**Published:** 2020-03-25

**Authors:** Morgan P. Kelley, Ivan A. Popov, Julie Jung, Enrique R. Batista, Ping Yang

**Affiliations:** Theoretical Division, Los Alamos National Laboratory, Los Alamos, NM 87545 USA

**Keywords:** Chemical bonding, Organometallic chemistry, Computational chemistry

## Abstract

In all known examples of metal–ligand (M–L) *δ* and *φ* bonds, the metal orbitals are aligned to the ligand orbitals in a “head-to-head” or “side-to-head” fashion. Here, we report two fundamentally new types of M–L *δ* and *φ* interactions; “head-to-side” *δ* and “side-to-side” *φ* back-bonding, found in complexes of metallacyclopropenes and metallacyclocumulenes of actinides (Pa–Pu) that makes them distinct from their corresponding Group 4 analogues. In addition to the known Th and U complexes, our calculations include complexes of Pa, Np, and Pu. In contrast with conventional An–C bond decreasing, due to the actinide contraction, the An–C distance increases from Pa to Pu. We demonstrate that the direct L–An *σ* and *π* donations combined with the An–L *δ* or *φ* back-donations are crucial in explaining this non-classical trend of the An–L bond lengths in both series, underscoring the significance of these *δ*/*φ* back-donation interactions, and their importance for complexes of Pa and U in particular.

## Abstract (web summary)

Metal-ligand *δ* and *φ* interactions, though considered weak, may be necessary for fully describing the electronic and geometric structures of certain compounds. Here, in actinide metallacycles, the authors discover two new types of M-L *δ* and *φ* back-bonds that contribute substantially to their unusual chemical behavior.

## Introduction

**Fig. 1:**
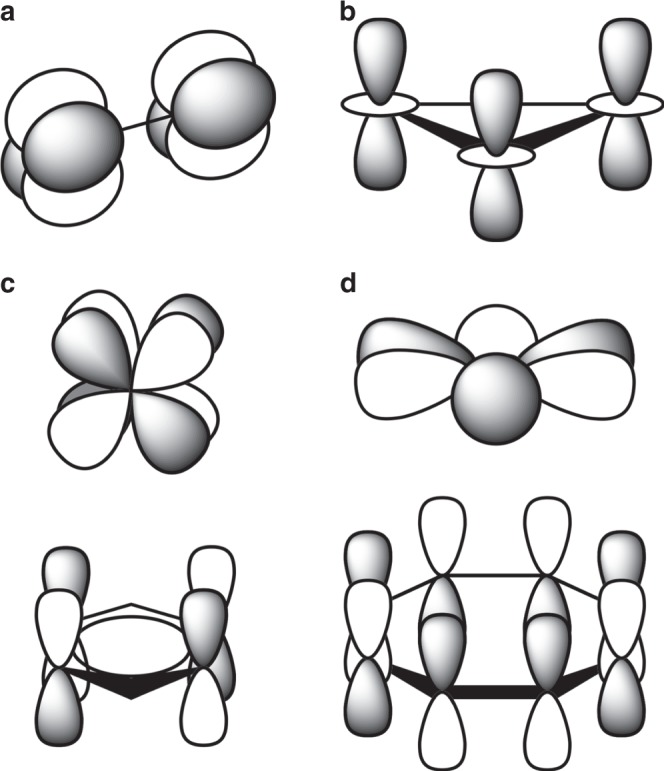
Schematic representation of AOs comprising *δ* and *φ* bonds. **a** M–M *δ* interaction between two *d*_*xy*_ (or ${{d}}_{{{x}}^2 - {{y}}^2}$) AOs. **b** M–M–M *δ* interaction among three ${{d}}_{{{z}}^2}$ AOs. **c** “head-to-head” M–L *δ* interaction between *f*_*xyz*_ (or ${{f}}_{{{y}}({{x}}^2 - {{z}}^2)}$) AO of metal (top) and unoccupied ligand orbital (bottom). **d** “side-to-head” M–L *φ* back-donation interaction between ${{f}}_{{{x}}({{x}}^2 - 3{{y}}^2)}$ (or ${{f}}_{{{y}}(3{{x}}^2 - {{y}}^2)}$) AO of metal (top) and unoccupied ligand orbital (bottom).

Due to the availability of *d*-electrons and *f*-electrons, chemical bonding in transition metal, lanthanide, and actinide compounds is more diverse and intricate compared to the compounds of the main group elements. Aside from classical *σ* and *π* interactions^1,2^, such systems may exhibit more exotic bonding modes, such as *δ*^3–11^ and even *φ*^12^. Most common examples of compounds exhibiting these bonds are those with direct metal–metal (M–M) contacts. Examples include various dimetals, M_2_, either bare or surrounded by stabilizing ligands, with multiple M–M bonds^3–11^. A typical *δ* bond reported in such systems features two nodal planes passing through the M–M axis due to the overlap of two *d*-type atomic orbitals (AOs), i.e., *d*_*xy*_ or $d_{x^2 - y^2}$ (Fig. 1a).

For more than two metal atoms (M_*n*_, *n* > 2), another type of the *δ* bonding can be achieved through interaction of *d*_*z*_^*2*^ AOs (Fig. 1b). In such cases, the *δ* bond features two parallel nodal planes located above and below the plane of the metal atoms, thus giving rise to *δ* aromaticity^13–15^. In contrast to the M–M *δ* bonds, metal–ligand (M–L) interactions involving a formation of *δ* bonds are less common. These bonds qualitatively differ from the M–M *δ* bonds for several reasons: first, they are formed due to the covalent overlap between occupied *d* or *f* metal orbitals and unoccupied ligand orbital(s), and, hence, are called *δ* back-bonds;^16–26^ second, such M–L interactions occur with “head-to-head” orbital overlap (Fig. 1c). The M–L *φ* back-bonds are even rarer^12^. They are formed due to the covalent overlap between occupied *f* metal orbital(s) and unoccupied ligand orbital(s) in a “side-to-head” fashion (Fig. 1d). To the best of our knowledge, there is only one study showing M–L *φ* bonding in actinocenes with the experimental verification of such interaction via the carbon K-edge X-ray absorption spectra (XAS)^12^.

Although the M–L *δ* or *φ* back-bonding is commonly considered a weak interaction, it may be as necessary as *σ* and *π* bonding interactions for the full description of electronic structure. The M–L *δ* back-bonding has been found to be instrumental in explaining certain features of several compounds^16–26^. For example, considering classical sandwich complexes of actinides, it was previously noted for cycloheptatrienyl sandwich compounds An(*η*^7^-C_7_H_7_)_2_ (An=Th–Am) that the *f*_*δ*_ orbitals not only participate in bonding via the e_2_″ *p*_*π*_ orbitals of the C_7_H_7_ rings, but are as essential as the *d*_*δ*_ orbitals in stabilizing the frontier *p*_*π*_ orbitals of these ligands^19^. Similarly, *δ* back-bonding was observed in tetravalent Pu^20^ sandwich complex with larger organic rings C_8_H_8_ (COT). It was theorized that the *δ* back-bonding in Pu(COT)_2_ structure may play a role in the observed migration of silyl substituents to different positions on the COT ligands^20^. Considering inverted sandwich complexes of actinides, it was earlier shown that the *δ* bonds formed between toluene molecule and two bridging uranium bis-amido fragments in (*μ*-C_7_H_8_)[U(N[R]Ar)_2_]_2_ (R=C(CH_3_)_3_, Ar=3,5-C_6_H_3_-Me_2_) make the unusual oxidation state of +2 accessible for the U center^21^. In a similar arene-bridged diuranium complex (*μ*-C_7_H_8_)U_2_(N[^*t*^Bu]Ar)_4_ (Ar=3,5-C_6_H_3_Me_2_), observation of the intense *f*−*f* bands was suggested to be due to the significantly covalent interaction between the U centers and the bridging toluene ligand supported by the presence of two *δ* bonds^22^. In other inverted sandwich complexes M_2_-2_2_-*μ*-C_10_H_8_ (M=Na, K) and 2_2_-*μ*-C_8_H_8_, *δ* bonding was invoked to explain the appreciably longer U−C_arene_ bond lengths in the latter complex (2.822 Å) in comparison to the former (2.634 Å), wherein a better covalent overlap was found^23^. Several other studies have also pointed to the need of using *δ* back-bonding for a comprehensive description of the M–L interaction between the divalent or trivalent U atoms and aromatic hydrocarbons^17,24–26^. In these structures, the U atom is bound equatorially to three O atoms of the scaffolding ligand, and axially to the arene allowing for a formation of two singly occupied *δ* bonds. Recently, a novel complex [((^Ad,Me^ArO)_3_*mes*)U(O)(THF)] was found to feature U–arene *δ* bonding^18^. It was suggested that the direct electronic communication between the U center and mesitylene moiety through a *δ* bond across all involved oxidation states of the catalytic cycle is important to enable M–L cooperative redox catalysis. Theoretical calculations supported a U(V) center with *δ* back-bonding to the mesitylene moiety, confirming [((^Ad,Me^ArO)_3_*mes*)U(O)(THF)] to be the first U(V) monoarene complex. A recent spectroscopic and theoretical study of actinocenes confirmed the presence of the M–L *φ* back-bonding in (C_8_H_8_)_2_U, although the 5f-*φ* mixing with the ligand orbitals was found to be minor^12^. Specifically, the bonding 1e_3u_ orbitals of (C_8_H_8_)_2_U were almost entirely metal based (6% C 2p, 94% U 5 *f*), while the antibonding 2e_3u_ orbitals were comprised mostly of ligand character (89% C 2p, 7% U 5 *f*). Considering all these examples, it is hard to overstate the importance of the *δ* and *φ* bonding for the description of electronic and geometric structures of actinide compounds.

**Fig. 2:**
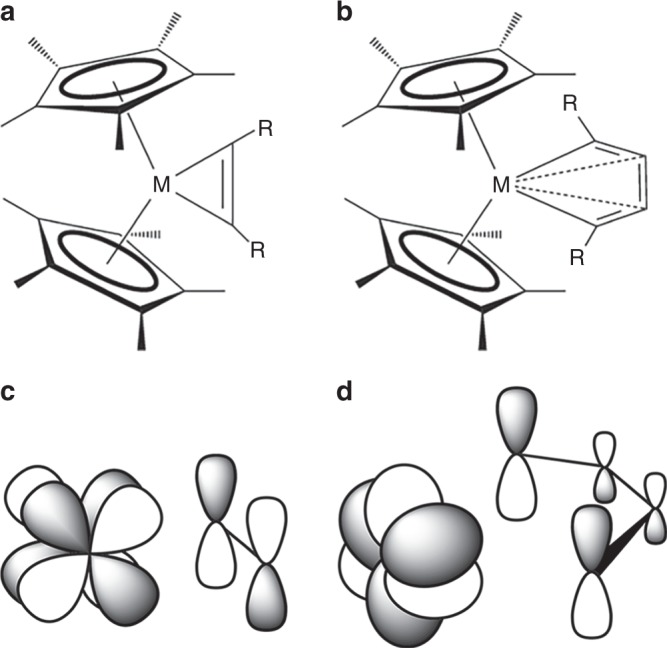
Actinide and transition metal metallacycle structures and their respective M–L *δ* and *φ* interactions. **a** Metallacyclopropene. **b** Metallacyclocumulene, (R = trimethylsilyl, phenyl; M = Ti, Zr, Th, Pa, U, Np, Pu). **c** “head-to-side” M–L *δ* interaction between *f*_*xyz*_ (or ${{f}}_{{{y}}({{x}}^2 - {{z}}^2)}$) AO of metal and unoccupied orbital of the cyclopropene ligand in (*η*^5^-C_5_Me_5_)_2_An[*η*^2^-C_2_R_2_]. **d** “side-to-side” M–L *φ* interaction between ${{f}}_{{y}{z}^2}$ (or ${{f}}_{{x}{z}^2}$) AO of metal and unoccupied orbital of the cyclocumulene ligand in (*η*^5^-C_5_Me_5_)_2_An[*η*^4^-C_4_R_2_], An=Pa–Pu.

It is worth emphasizing that in all actinide complexes reported thus far, the metal center(s) is not in the same plane(s) of ligand atoms and the M–L back-bonding is formed exclusively by the “head-to-head” *δ* or “side-to-head” *φ* interactions of either singly or doubly occupied *f* AOs of the metal and unoccupied orbital(s) of the stabilizing ligand (Fig. 1c, d). This begs the question of whether other types of such bonding modes are possible when the metal center is in the same plane of ligands. In this report, we show for the first time two novel types of the M–L back-bonding in actinide metallacyclopropenes (*η*^5^-C_5_Me_5_)_2_An[*η*^2^-C_2_(SiMe_3_)_2_] and metallacyclocumulenes (*η*^5^-C_5_Me_5_)_2_An[*η*^4^-C_4_(SiMe_3_)_2_] for the actinide series from Th to Pu, i.e., “head-to-side” *δ* and “side-to-side” *φ* M–L interactions, respectively (Fig. 2c, d). These two unique bonding modes are made possible by a number of factors. First, there is a smaller number of C atoms in the ligand interacting with the metal center (2 in metallacyclopropenes and 4 in metallacyclocumulenes) than in any previously reported complexes featuring M–L *δ* or *φ* back-bonding, where this number ranged from 6 to 16. Second, availability of *f* electrons is a necessary requirement for the formation of such bonds, making them distinct from transition metal containing complexes, as well as from actinides without *f* electrons. Third, positioning of the ligand C atoms of the propene and cumulene ligands in plane with the metal center allows for specific interactions of 2*p* orbitals of the C atoms with the 5*f* orbitals of the metal center, making them different from any other systems featuring M–L *δ*/*φ* back-bonds where the M center is located out of the plane of the ligand atoms (Fig. 1c, d).

Based on recent syntheses reports^27–32^ and our chemical bonding models for Th and U complexes of metallacyclopropenes and metallacyclocumulenes, we predict three more actinide compounds of Pa, Np, and Pu, which also help to fully understand the electronic structures of the Th and U complexes. Specifically, we demonstrate that the direct L–M *σ* and *π* donations combined with the M–L *δ* or *φ* back-donation are crucial in explaining the non-classical trend of the M–L bond lengths in both series, thus underscoring the significance of these *δ* and *φ* interactions. The unique bonding features of these actinide metallacycle systems expand our knowledge of organo-actinide chemistry in general, and are likely to have a measurable impact on the structures and interactions in similar actinide compounds.

## Results

### Geometry

Our DFT optimized geometries for the metallacyclopropene and metallacyclocumulene complexes (henceforth referred to as ‘propene’ and ‘cumulene’, respectively, for brevity) match published crystal structures, with bond distances and angles generally within experimental uncertainty for both Group 4 transition metals^33–39^ and actinides (Th and U);^27–32^ see Supplementary Tables 1, 2. The calculated IR spectrum matches the measured spectra of U-cumulene, including the strong C_α_–C_β_ stretch peak at 1590 cm^–1^ discussed in the literature^32^. Calculated IR spectra are given in Supplementary Fig. 1.

**Fig. 3:**
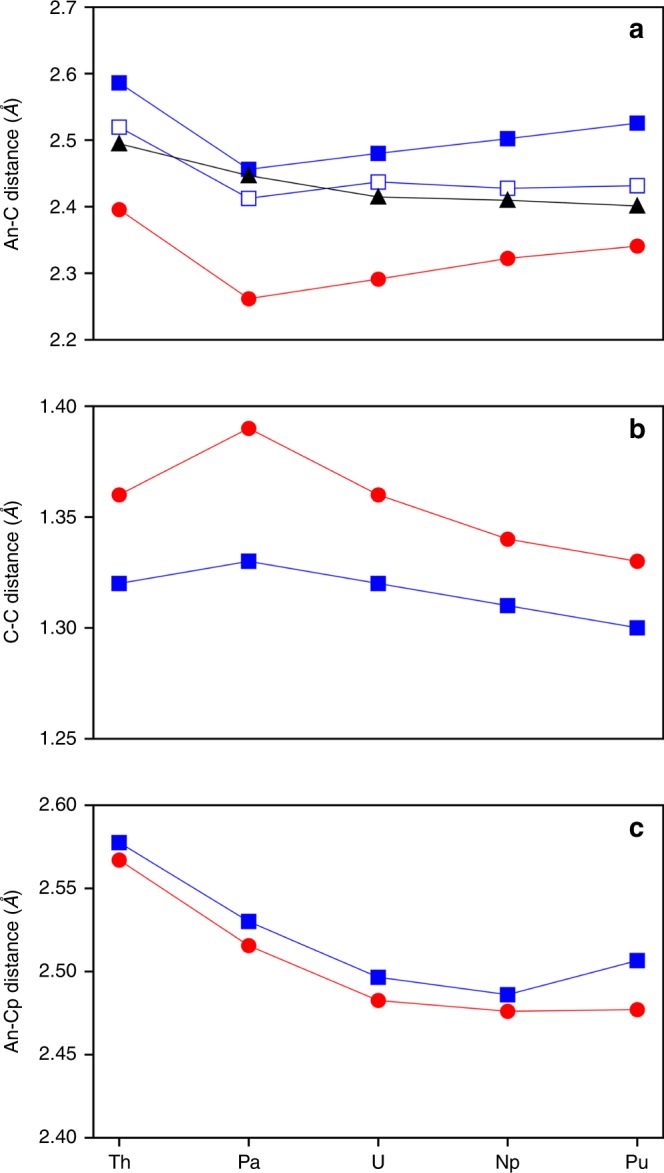
Selected geometric distances in propene and cumulene complexes. An–C (**a**), C–C (**b**), and An–Cp (**c**) distances for propenes (red circles), cumulenes (blue squares), and (C_5_Me_5_)_2_An(CH_3_)_2_ (black triangles). M–C_α_ and M–C_β_ distances are shown as filled blue and hollow blue squares for the cumulene series, respectively. C_α_–C_β_ distances are shown for cumulenes. An–Cp distances are given as distances between the An ion and Cp ring centroid. Values (in Å) are reported as averages; standard deviations are not significant. The substituent R group was not found to strongly affect these distances (Supplementary Tables 1, 2); for clarity, only those for R = trimethylsilyl are shown.

Fig. 3 shows distance trends in relevant M–C, M–Cp, and C–C distances for calculated structures across the An series. It is quite surprising that in spite of the contracting ionic radii of the actinide ions^40^, the M–C distances in the propene series (red circles in Fig. 3a) increases from Pa (2.26 Å) to Pu (2.34 Å) for both the trimethylsilyl and phenyl substituents. The M–propene distances for the Th complexes, however, are longer than those of any other complex; this is consistent with published crystal structures for Th-propene^27^ and U-propene^28^, which show the U–C bonds to be approximately 0.1 Å shorter than the corresponding Th–C bonds (Supplementary Table 1). Indeed, the M–propene and M–cumulene distances are clearly not decreasing from Th to Pu as expected due to the actinide contraction. This trend is opposed to that of the M–Cp distances (Fig. 3c), which generally decrease across both series. This hints that some special interactions taking place between the metal center and propene/cumulene ligands. Interestingly, the C–C distances in the propene structures follow an almost opposite trend to that of the M–C bonds, with an increase from Th (1.36 Å) to Pa (1.39 Å) preceding a consistent decrease from Pa to Pu (1.33 Å).

In the cumulene complexes, the metal center interacts with all four cumulene C atoms, with the C_β_ atoms (the interior two carbon atoms in the cumulene) being slightly closer than their C_α_ (the peripheral cumulene carbon atoms connected to the R groups; Fig. 2b) counterparts. It is worth noting that the cumulene M–C_α_ distances follow the same trend as seen in the M–C distances of the propene series, with an approximately 0.1 Å drop in distance between Th and Pa followed by a consistently increasing distance across the remainder of the series from Pa to Pu (filled blue squares in Fig. 3a). However, this trend is not replicated in the M–C_β_ distances (hollow blue squares in Fig. 3a), which—apart from the drop between Th and Pa—remain fairly constant across the series, thus suggesting that it is the M–C_α_ interactions that dominate the M–C bonding. Similar to the propene complexes, the trends between Th and U are reflected in published crystal structures (Supplementary Table 2)^29–32^. Comparing experimental crystal structures one can see that there is an overall increase in the M–C distances by approximately 0.2 Å in the cumulene complexes relative to their propene counterparts. Peripheral C–C bond distances (C_α_–C_β_) increase from Th—where they are similar to transition metal complexes with Ti and Zr—to Pa, and then decrease across the series to Pu, matching the C–C trend in the propene complexes. Meanwhile, the central (C_β_–C_β_) distances remain essentially constant across the series, differing by less than 0.01 Å, indicating some differences in the bonding interactions of the metal ion with the C_α_ and C_β_ atoms.

Based on the two points available in the literature for actinides (Th and U)^27–32^, it is difficult to predict a priori the unusual trend of the M–C bond distances as a function of the metal ion (Th–Pu) in both the propene and cumulene series. One may assume that the specificity of the M–C bond distances is due to the possible delocalized M–L bonding interactions, which occur due to the presence of *π* electrons in the C_2_R_2_ (C=C *π* system) and C_4_R_2_ (C=C=C=C *π* system) ligands. Indeed, in the simple (C_5_Me_5_)_2_An(CH_3_)_2_ system the trend is classical. In these systems the propene or cumulene ligands are replaced by two separate methyl groups forming two single An–C bonds, and no electron delocalization or donation between these ligands and the metal is anticipated except for the direct *σ* bonding. As one would expect from the An contraction^40^, the M–CH_3_ distances steadily decrease from Th (2.49 Å) to Pu (2.40 Å) (black triangles in Fig. 3a). To understand the reasons of the unusual non-classical M–C bond length trend in both the propene and cumulene series, as well as to explain other structural trends in these complexes, we turn to an in-depth analysis of their electronic structure and bonding.

### Chemical bonding analysis: canonical molecular orbitals

**Fig. 4:**
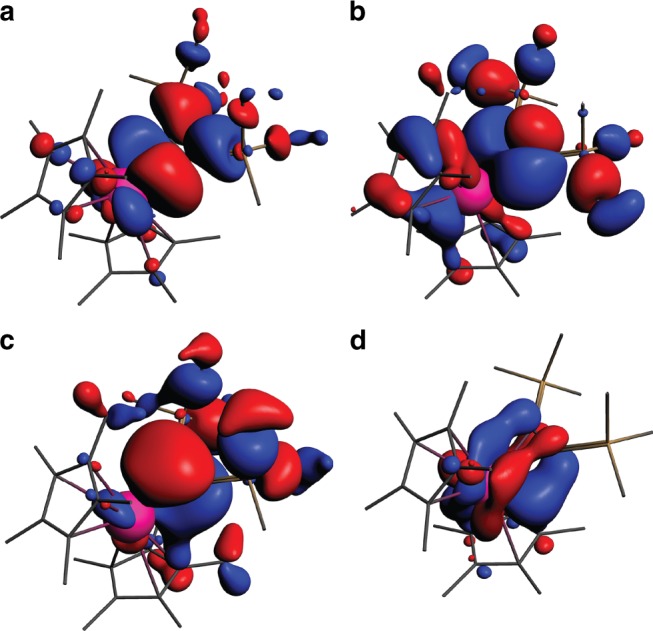
Selected CMOs of the U metallacyclopropene with trimethylsilyl groups. **a** HOMO-2, *σ* (**b**) HOMO-7, *σ* (**c**) HOMO-8, *π* (**d**) HOMO-1 (SOMO), *δ*. H atoms are omitted for clarity. ISO = 0.025.

While the strength of M–L interactions is primarily dominated by *σ* bonding, *π* contributions are of no less importance. Indeed, together *σ* and *π* interactions are the main descriptors of bonding in many actinide systems, and sufficient to describe their electronic and geometric structures^1,2^. As reported previously^41–45^, bonding between the metal center and the propene or cumulene ligands in similar metallacycle complexes of Group 4 transition metals occurs through two M–C *σ* bonds and one C–C *π* bond donating electron density to the metal center. For actinide metallacycles, previous computational studies on Th and U complexes reported these two types of bonds with the *σ* bonds noted as being composed of hybrid 6*d*-5*f* An orbitals^27,28^. Our calculations of the canonical molecular orbitals (CMOs) also found the reported *σ* and *π* interactions (Fig. 4a-c). More importantly, we identify the existence of the *δ* orbital in An-propene complexes (Fig. 4d) and the *φ* orbital in An-cumulene complexes (Supplementary Fig. 12c), both critical to full understanding of the electronic structure of these complexes. Indeed, these orbitals are absent in Th, which has no 5*f* electrons, but do appear in Pa, U, Np, and Pu at or near the HOMO level as a singly occupied orbital composed primarily of An 5*f*. These *δ/φ* orbitals are strongly polarized towards the metal, with increasing polarization as the series is traversed (Supplementary Figs. 2, 3). They are particularly noteworthy due to the “head-to-side” *δ* and “side-to-side” *φ* M–L back-bonding, wherein the 5*f* orbital of actinide interacts with the “sides” of 2*p* orbitals of C atoms (Fig. 2c, d).

Due to the complexity of the CMOs, which are intrinsically difficult to interpret because they tend to be delocalized, we have utilized the Adaptive Natural Density Partitioning (AdNDP) analysis^46^. It allows transformation of all delocalized CMOs into more localized bonding elements (*n* center, two electron (*n*c–2e) objects), which are more chemically intuitive and easier to interpret in terms of chemical bonds. The AdNDP analyses provides necessary information about the presence and type of bonds on every fragment of the system, as described in the following sections. The occupation number, ON, which is the number of electrons occupying a particular identified localized state, serves as the indicator of a bond strength. Due to the similarities in bonding between the two series, the propenes will be discussed in detail followed by a discussion of cumulenes highlighting their differences.

### Localized representation of chemical bonding in propenes

In total, there are 86 valence electron pairs in the (*η*^5^-C_5_Me_5_)_2_Th[*η*^2^-C_2_(SiMe_3_)_2_] complex with the additional 1, 2, 3, and 4 unpaired *f*-electrons on Pa, U, Np, and Pu, respectively. According to the AdNDP electron density partitioning scheme, these singly and doubly occupied delocalized CMOs are transformed into the *σ*, *π*, and *δ* bonds as outlined below.

**Fig. 5:**
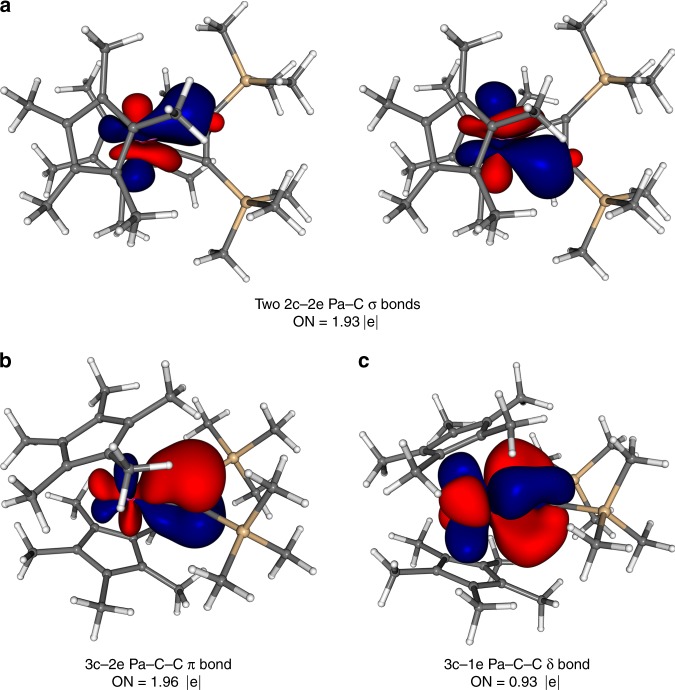
AdNDP bonding elements found between the C_2_(SiMe_3_)_2_ ligand and the M center in the example of Pa-propene complex. **a** Two direct Pa–C *σ* bonds (left and right). **b** Three-center Pa–C–C *π* bond. **c** Singly occupied three-center Pa–C–C *δ* bond. Sticks between atoms help visualization and do not necessarily represent classical 2c–2e bonds here and elsewhere. ON is equal to 2.00|e| or 1.00|e| in an ideal case for a doubly or singly occupied bond, respectively.

*σ* bonding: AdNDP identifies 50 two-center two-electron (2c–2e) covalent *σ* bonds (C–C and C–H *σ* bonds) in the two C_5_Me_5_ fragments (Supplementary Fig. 4a, b) and 27 2c–2e *σ* bonds on the alkyne ligand C_2_(SiMe_3_)_2_ (Supplementary Fig. 4c, d), all with high ON values in the range of 1.93–1.99|e|. The remaining four *σ* electrons are found as two direct 2c–2e *σ* bonds connecting the An atom with two C atoms of the alkyne fragment (Fig. 5a). These two bonds originate from the two CMOs (HOMO-2 and HOMO-7 in the example of the U complex, Fig. 4a, b), and differ from all other 2c–2e *σ* bonds since they are highly polarized towards C atoms, with 75–81% of the electron density coming from the C atoms.

**Fig. 6:**
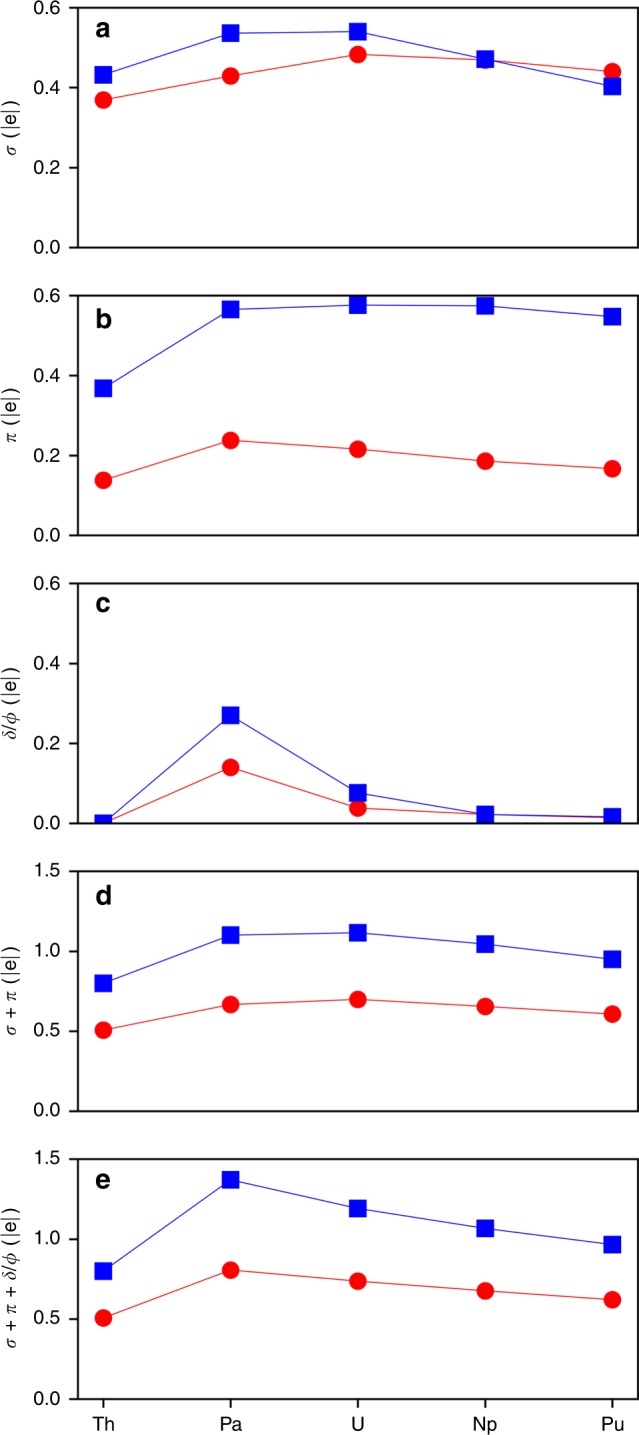
Magnitude of the direct L–M *σ* and *π* donations and M–L *δ* or *φ* back-donations between the C_2_(SiMe_3_)_2_ (propene series (*δ*), red circles) and C_4_(SiMe_3_)_2_ (cumulene series (*φ*), blue squares) ligands and the M center in the Th–Pu series. **a** L–M *σ* donation. **b** L–M *π* donation. **c** M–L *δ/φ* back-donation. **d** L–M (*σ* + *π*) donation. e Overall (*σ* + *π* + *δ/φ*) donation.

In agreement with previous studies^27,28^, the M–C *σ* bonds are primarily formed by the interaction of 6*d*-5*f* hybrid orbitals of the M with the in-plane 2*s*-2*p* hybrid orbitals of the C atoms (Fig. 5a, Supplementary Figs. 6, 7). While for all the An atoms *d*-character of the M hybrid orbitals is prevalent over *f*-character, the fraction of 5*f* substantially increases from Th to Pu (11.29 to 36.82%), which underlines the increasing role of 5*f* electrons in the M–C *σ* bonding as the An series is traversed. The *d*-electron density comprising these bonds is highest for the Th complex (73.56%), decreasing across the series to Pu (44.49%). It is important to note that the M–C *σ* bonds revealed in this series are found to have quite high ON values (1.92–1.93|e|), and their magnitude stays nearly the same along the series (Supplementary Fig. 8). The contribution of the M center in the M–C *σ* bonding is found to be the highest for U (0.48|e|) and the smallest for Th (0.37|e|) (Fig. 6a, Supplementary Table 3). As one would expect, the higher L–M donation to the metal center should correspond to the shorter L–M bond distance. However, the shortest M–C bond is found in the Pa complex, which does not show the highest L–M donation (0.43|e|). This observation hints at other important interactions impacting the structure of these complexes, as discussed below.

*π* bonding: As opposed to the strong 2c–2e C–C *σ* bonding within the C_2_(SiMe_3_)_2_ fragment (ON = 1.90–1.98|e|), the 2c–2e C–C *π* bonding of the propene ligand was shown to have lower ON values (1.72–1.82|e|), indicating a possible delocalization over the larger number of centers (Supplementary Fig. 9a). Per AdNDP, the M center is involved in the formation of the 3c–2e *π* bond with two C atoms of the alkyne fragment (Fig. 5b) that originates from the HOMO-8 in the case of the U complex (Fig. 4c). Similar to the M–C *σ* bonds, the ON values of the M–C–C *π* bonds do not change significantly within the series, i.e., ON = 1.95–1.96|e| (Supplementary Fig. 8), as opposed to the L–M *π* donation trend (Fig. 6b). This L–M *π* donation occurring from the 2*p*-dominant orbital of the C_2_(SiMe_3_)_2_ ligand into the unoccupied *d*-dominant (in the case of Th) or *f*-dominant (in the case of Pa–Pu) hybrid orbital of the An atom is in the range of 0.14–0.24|e|, or 7.04–12.13% of the total *π* electron density of the 3c–2e *π* bond. The combined (*σ* + *π*) L–M donation (Fig. 6d) shows the highest value for the U-propene interaction (0.70|e|) and the smallest value for the Th-propene bonding (0.51|e|). Thus, with the exception of Pa, the (*σ* + *π*) L–M electron donation explains the observed M–C geometrical changes. Similarly, excepting Pa, C–C bonds correlate with the L–M donation, with stronger donation corresponding to the weaker and thus longer bonds (Fig. 3b). Obviously, the Pa-propene bonding cannot be described only by the *σ* and *π* interactions, and other interactions are necessary to explain the shortest Pa–C bond distance.

It is worth noting that the *σ* and *π* chemical bonding elements found for the M–C_2_(SiMe_3_)_2_ interaction in these actinide complexes are qualitatively similar to those of Group 4 propenes^48^. The *π* electron donation resulting in the formation of the 3c–2e *π* bonds shows even larger magnitude compared to the Group 4 propenes (Supplementary Table 4), which were previously characterized as aromatic on the basis of the computed stabilizing energy and negative nucleus-independent chemical shifts indices^48^. This suggests the possibility of even stronger *π* electron delocalization over this fragment in actinides. Similar to the Group 4 propenes, the direct L–M *π* donation in Th complex occurs to the unoccupied *d*-orbital of the metal. In contrast to the Group 4 species, an additional interaction in actinide metallacycles—not identified previously—plays an important role in the electronic structure of these systems due the availability of 5*f*-electrons, particularly for the complex of Pa as discussed below.

*δ* bonding: While the strength of the interaction between the An atom and the alkyne fragment is primarily dominated by the strength of their *σ* and *π* bonding, these two interactions alone (Fig. 6d) do not fully explain the peculiar trend of the M–C distances observed in the An series (Fig. 3a). The combined (*σ* + *π*) L–M interactions can account for the M–C bond length increase from U to Pu, countering the actinide contraction^40^. However, an additional M–L *δ* back-bonding (Fig. 6c) interaction, strongest in the case of the Pa complex, fully reconciles the donation trend with the M–C bond trend (Figs. 3a, 6e). The *δ* back-donation originating from the HOMO (SOMO) orbital of the Pa complex (HOMO-1 in the case of U, Fig. 4d) occurs via the promotion of electron density from the *f*_*xyz*_ orbital of the An atom to the antibonding π^*^-orbital of the C–C fragment of the C_2_(SiMe_3_)_2_ ligand (Fig. 5c, Supplementary Fig. 9b). This interaction results in the formation of a singly occupied *δ* bond comprised of the metal center and the two propene C atoms (3c–1e *δ* bond). This 3c–1e *δ* bond is the first example of the “head-to-side” M–L *δ* interaction occurring between the 5*f* orbital of the metal and π^*^ orbitals of the ligand with the metal center lying in the same plane of the ligand.

Except for Th, all other actinide metals examined here (Pa–Pu) have unpaired *f*-electrons, one of which has the correct symmetry to form a *δ* bond with the propene ligand (Supplementary Fig. 10). However, only Pa and U promote a non-negligible electron density into the antibonding ligand orbital of the propene, 0.14|e| and 0.04|e|, respectively. The *δ* back-bonding in Np and Pu is appreciably smaller— 0.02|e| and 0.01|e|. This is in agreement with the energy gap between the singly occupied *δ* orbital and corresponding unoccupied ligand orbital that is found to be smallest for Pa, with a constant increase to Pu (Supplementary Table 7). Indeed, while in the case of U the presence of the M–L *δ* back-donation does not impact the overall (*σ* + *π* + *δ*) donation trend, it does so in the case of Pa. Specifically, accounting for the M–L *δ* donation results in the highest overall donation value for Pa instead of U (Fig. 6e), in agreement with the shortest Pa-propene distance. Hence, the collective effect of all three interactions (*σ* + *π* + *δ*) counters the impact of the An contraction and produces a trend fully consistent with the peculiar trend of the M–C bond lengths across the An series (Fig. 3a). Likewise, populating the π^*^ orbital of the propene C atoms destabilizes the C–C bonding, in agreement with the oppose trend for C–C bond distances (Fig. 3b). In general, the effect of back-donation is also observed in many transition metal complexes containing CO ligands (e.g., Ti_3_(CO)_3_^49^), which are capable of accepting *π* electron density from the metal via the M–L *π* back-donation, thus weakening the C≡O vibrational mode^50^.

The most pronounced M–L *δ* bonding interaction in the Pa complex among other actinides within the propene series is in excellent agreement with the previous studies of diatomic actinides indicating the enhanced role of the 5*f* orbitals of Pa compared to U in the formation of the *δ* bonds, which are stronger in the dimer molecule Pa_2_ than in U_2_, thus leading to the effective bond orders of 4.5 and 4.2, respectively^,8,11^.

### Chemical bonding in metallacyclocumulenes

Due to the qualitative similarities in the geometrical structures, as well as in the trends (bond lengths, angles) of the propene and cumulene complexes (Fig. 3), chemical bonding features of these compounds are found to be similar, though with some alterations (see Supplementary Discussion for the complete AdNDP analysis of cumulenes). In brief, the main difference is seen in the larger number of bonding interactions, as well as in a more delocalized bonding pattern due to the larger number of C atoms constituting the cumulene ligand. For instance, two direct M–C_α_ *σ* bonds (ON = 1.65–1.69|e|) (Supplementary Fig. 14a) can also be viewed as two 3c–2e *σ* bonds involving an additional C_β_ atom (ON = 1.94–1.95|e|). This explains the appreciable elongation of the M–C bonds in the cumulene vs. the propene series (2.46–2.59 Å vs. 2.26–2.40 Å, respectively).

The similarity between the cumulenes and propenes extends to their *π* bonding as well, though the *π* interactions are slightly altered. Instead of just one 3c–2e *π* bond present in propenes, there are three *π* L–M interactions: one central M–C_β_–C_β_ (considered as *σ* with respect to the metal center) and two peripheral M–C_α_–C_β_ *π* bonds (Supplementary Figs. 14b, 19). Similar to the propenes, analyzing only the (*σ* + *π*) bonding interactions does not fully explain the peculiar trend of the M–C distances observed in the An series. Due to the additional C atoms of the cumulene ligand, the more delocalized 5c–1e *φ* bond (Supplementary Figs. 14c, 21) is formed in lieu of the 3c–1e *δ* bond found in propenes. It is worth noting that the back-donation is approximately twice as strong in cumulenes as it is in propenes (Fig. 6c, Supplementary Table 3). As in propenes, the M–L *φ* back-bonding is strongest in Pa (0.27|e|); however, the *φ* interaction of U (0.08|e|) cumulene is significantly stronger than that of Np (0.02|e|) or Pu (0.02|e|). Although the U–propene *φ* back donation is still considered as a minor effect since it does not significantly impact the overall donation trend, it cannot be discounted. As a matter of fact, its presence is verified experimentally as discussed below. In contrast, the Pa–propene *φ* back-bonding does impact the overall (*σ* + *π* + *φ*) donation trend (Fig. 6e), showing that the Pa complex features the highest collective M–L donation in the series. Overall, presence of the M–L *φ* interactions in the cumulenes supports the observed M–C bond length trend, thus confirming its indispensable role in explaining the peculiar geometrical changes found in the cumulene series.

### Optical properties

UV-visible and near-IR spectroscopy are among the most used experimental tools to probe chemical bonding interactions^3,4,9,17,18,22,24–26,34,39,51–53^. The UV-visible-NIR spectra for the cumulene complex (C_5_Me_5_)_2_U[η^4^-1,2,3,4-PhC_4_Ph] has been recently reported^32^, providing an opportunity for experimental validation of the orbital analysis discussed above. In particular, the appearance of transitions involving the *φ* bonding orbital would allow this interaction to be measured experimentally. Though the reported U cumulene spectra is composed of only a single broad peak in the UV-visible region, several features (appearing as shoulders in the single peak) can be identified through calculation of its 1st and 2nd derivatives. The near-IR region is better defined, and exhibits several peaks. Both regions provide evidence for the existence of the *φ* bonding orbital.

**Fig. 7:**
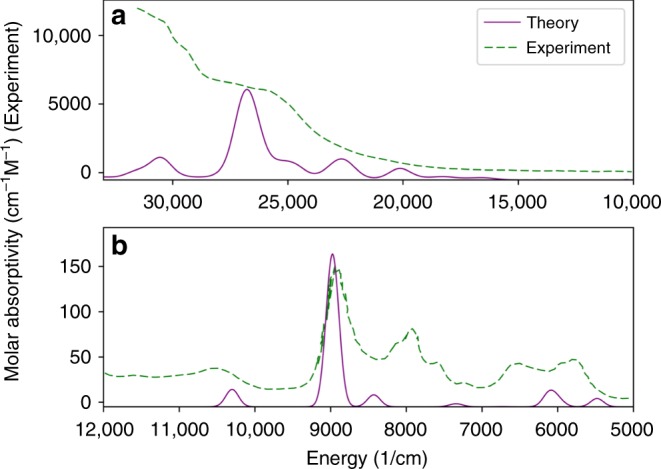
UV-Visible-NIR electron absorption spectra for (C_5_Me_5_)_2_U[η^4^-1,2,3,4-PhC_4_Ph]. Experimental values (dashed lines) digitized from ref. ^32^. **a** UV-Visible range. **b** Near IR range. Peaks at 8,936 cm^–1^ and 20,625 cm^–1^ with oscillator strengths of 0.0102 and 0.0387, respectively, are derived from transitions involving the *φ* bonding orbital.

Natural transition orbitals (NTO) allow the visualization of transitions as single electron excitations from one orbital to another^54^, and the calculated absorption spectra of the (C_5_Me_5_)_2_U[η^4^-1,2,3,4-PhC_4_Ph] complex matches with experimental measurements reasonably well (Fig. 7). Of primary interest in this discussion are two peaks, occurring in the experimental spectrum at 8,936 cm^–1^ and 20,625 cm^–1^ with oscillator strength values of 0.0102 and 0.0387, respectively. The first of these peaks, in the near-IR region, is easily separated from surrounding peaks, though the second is visually obscured in the tail of the experimental spectrum and was only identified through analysis of the 2nd derivative. The peak at 8,936 cm^–1^ is composed of a single NTO transition from the *φ* bonding orbital (HOMO-1 for the U complex) to a virtual orbital comprised of C–C π^*^ interactions and localized U 5*f*, providing the first concrete experimental evidence for the *φ* interaction. The peak at 20,625 cm^–1^ is less clear-cut, being comprised of 3 NTO transitions, the least of which (11%) is from the bonding *φ* orbital to a virtual orbital comprised primarily of C–C π^*^ interactions with a small contribution from a 5*f* orbital. It is worth noting that the calculated intensity of both of these peaks changes in tandem with the strength of the *φ* bond in the An series (Supplementary Figs. 22, 23), providing a simple experimental tool to assess its strength. The oscillator strength of single NTO transition in the near-IR region of the Pa complex is appreciably higher than that of U (0.0164 vs. 0.0102), in line with Pa having the strongest M–L *φ* back-bonding interaction in the series.

### Reactivity

It was suggested previously^27,28,47^ that the more covalent character of the bonding between U and the two C atoms of the C_2_(SiMe_3_)_2_ ligand in the propene complex causes a reactivity pattern that is different from that of the Th complex, which exhibits more ionic M–C bonding. Specifically, (*η*^5^-C_5_Me_5_)_2_U[*η*^2^-C_2_(SiMe_3_)_2_] may act as a U(II) synthon (source of (*η*^5^-C_5_Me_5_)_2_U(II)) when reacted with alkynes, as opposed to the (*η*^5^-C_5_Me_5_)_2_Th[*η*^2^-C_2_(SiMe_3_)_2_] counterpart, which shows no reactivity towards alkynes^28,47^. Based on this, on can expect that the reactivity of the Pa complex, exhibiting the smallest NPA charge on the metal center (+1.38) in the propene series (Supplementary Fig. 5), should be similar to that of the U (+1.79) complex and opposite to those of Th (+2.12), Np (+2.35), and Pu (+3.04) which are all expected to have more ionic An–C bonding. The corresponding values for the Ti (+1.78) and Zr (+1.62) complexes are also close to the U counterpart, in agreement with previous studies showing the ability of Group 4 transition metal complexes to participate in the substitution reactions with alkynes^44,55,56^. Similar to propenes, the NPA charge on the metal center of the Pa cumulene complex suggests that its reactivity towards alkynes should be comparable to that of the recently synthesized (*η*^5^-C_5_Me_5_)_2_U[*η*^4^-C_4_(SiMe_3_)_2_], as well as Group 4 cumulenes, whereas the reactivity of the corresponding Np and Pu complexes should be similar to the Th complex^30,31^ (Supplementary Fig. 15).

## Discussion

In this paper we introduced two types of chemical bonding between an actinide metal and two different ligands, the “head-to-side” *δ* and “side-to-side” *φ* M–L back-bonds found in the actinide complexes of metallacyclopropenes and metallacyclocumulenes, respectively. Due to the relatively small number of C atoms in the ligands interacting with the metal center in the same plane that allows for their specific positioning, the unique bonding modes become possible via the unusual interaction of the 5*f* orbitals of the metal with the “sides” of 2*p* orbitals of the C atoms. Availability of *f* electrons makes them distinct from the corresponding transition metal containing complexes, as well as from actinides without *f* electrons. In addition to the known Th and U complexes of propenes and cumulenes, we predicted three novel complexes of Pa, Np, and Pu. We demonstrated that, with the exception of Pa, the direct L–M (*σ* + *π*) electron donations explain the non-classical trend of the M–propene and M–cumulene bond lengths (Th–Pu). One additional interaction, the M–L *δ/φ* back-bonding, which is found to be the strongest in the case of the Pa complexes, helps to make the donation trends fully consistent with the unusual M–C bond trends. This shows that the collective effect of all three interactions (*σ* + *π* + *δ/φ*) counters the impact of the An contraction and underscores the importance of the *δ/φ* bonding modes for the predicted complexes of Pa in particular, where they have a comparable effect to the L–M *π* bonding interactions.

Although the U–propene *δ* and U–cumulene *φ* back-bonding is considered to be only a minor effect, as it does not impact the overall donation trend, it cannot be discounted. We have shown how this M–L *φ* interaction can be assessed experimentally in the example of the recently synthesized (C_5_Me_5_)_2_U[η^4^-1,2,3,4-PhC_4_Ph] complex. In addition, if the (C_5_Me_5_)_2_Pa[η^4^-1,2,3,4-PhC_4_Ph] complex is synthesized in the future, it is expected to see an increased strength of the *φ* interaction in the UV-vis-NIR spectrum. In general, these M–L *δ/φ* back-bonding interactions can also be seen in other actinide complexes and hold potential implications for nuclear separation chemistry. Presence of *f* electrons available for such interactions can enhance the M–L bonding and, hence, increase separation efficiency for actinides as compared to the corresponding lanthanide complexes, which generally have very localized *f* electrons.

## Methods

### Density functional theory

Structures of the metal complexes were optimized using density functional theory (DFT) with the PBE functional^57^, scalar relativistic ZORA Hamiltonian, and triple-ζ plus two polarization function (TZ2P) basis sets with the frozen core approximation applied to the inner shells [1s^2^-4f^14^] for actinide atoms and [1s^2^] for the other atoms^58,59^. Effects on the molecular orbital energy levels from spin-orbit coupling were negligible (Supplementary Figs. 24–28). Geometry optimization and frequency calculations were performed using ADF 2016^60,61^. The PBE functional was found to well reproduce the experimental structural results of the complexes discussed here (Supplementary Tables 1, 2), characteristic IR spectra, and the features of the UV-Visible and NIR spectra of the U-cumulene complex (Fig. 7). Hence, PBE data was used throughout the manuscript. This is also consistent with the previous success by the PBE functional in studying complicated actinide compounds^62–64^. Since in general GGA functionals like PBE tend to over-delocalize electrons, potentially magnifying the effect of the *δ* and *φ* bonds discussed here, we also employed PBE0 hybrid functional. It was found that both *δ* and *φ* interactions become smaller at PBE0, although the trends across the An series are qualitatively comparable for both GGA and hybrid functionals, and both are fully consistent with the M–C bond distances (compare Fig. 6 with Supplementary Fig. 29). It is also worth noting that the PBE0 functional does not change the conclusions about the role of the M–L *δ* or *φ* interactions in both series, underscoring their roles in complexes of Pa in particular.

### Chemical bonding analysis

Chemical bonding analysis of the studied compounds was performed using the AdNDP method^46^. AdNDP analyzes the first-order reduced density matrix in order to obtain its local block eigenfunctions with optimal convergence properties for an electron density description. The obtained local blocks correspond to the sets of *n* atoms (*n* ranging from one to the total number of atoms in the molecule) that are tested for the presence of two-electron objects (*n*-center two-electron (*n*c–2e) bonds) associated with this particular set of *n* atoms. Thus, the AdNDP method recovers both Lewis bonding elements (1c–2e and 2c–2e objects, corresponding to the core electrons and lone pairs, and traditional 2c–2e bonds), as well as delocalized bonding elements (*n* > 2). The user-directed form of the AdNDP analysis can be applied to specified molecular fragments and is analogous to the directed search option of the standard natural bond orbital (NBO) code^65,66^. From this point of view, AdNDP achieves a seamless description of systems featuring both localized and delocalized bonding without invoking the concept of resonance. It accepts only those bonding elements whose occupation numbers (ONs) exceed the specified threshold values. Previously, AdNDP has been shown to be a very efficient and visual approach for the interpretation of the molecular orbital-based wave functions for various systems with complex bonding patterns^13,15^. AdNDP calculations were performed using PBE functional in conjunction with ECP60MWB_ANO basis set with the small core pseudopotentials. 6–311 G(d) basis was employed for all other atoms. Previously, AdNDP was shown to be insensitive to the level of theory or the basis set used^67^. In this paper, the functional and basis set dependency of AdNDP was tested for both actinide metallacycle series. It was found that the qualitative picture of the M–L donation trends in both series from Th to Pu stays the same at both PBE and PBE0 functionals (Supplementary Fig. 29). In addition, as long as the basis set is large enough to sufficiently describe electronic structure, AdNDP showed no dependency on the basis set used (Supplementary Table 8). The density matrix was obtained from the NBO5.9 calculations using Gaussian 09 software package^68^. The choice of the NBO version (NBO5.9 vs. NBO6.0) was not found to qualitatively affect the results of the AdNDP algorithm. The Molekel 5.4.0.8 program^69^ was used for molecular orbitals visualization of the AdNDP results.

### CASSCF calculations

In order to assess the multi-reference character of the electronic wave function in the investigated systems, and the consequence of such effects on the AdNDP bonding analysis, complete active space self-consistent field (CASSCF)^70^ calculations were carried out using the quantum chemistry package ORCA^71^. Because of the electronic structure similarities between the two series (cumulenes and propenes), and given that these calculations are very demanding of computational resources, we focused on the more complicated cumulene series for the CASSCF calculations. The conclusions drawn for the cumulene series are expected to hold for the propene series. In these calculations, scalar relativistic effects are included using the second order Douglas-Kroll-Hess Hamiltonian^72,73^. Spin-orbit coupling is added using a mean-field approach through quasi-degenerate perturbation theory^74^. Dynamical correlation is added through second order N-electron valence state perturbation theory (NEVPT2) method, without frozen core^75–77^. The convergence of the CASSCF calculations is achieved towards tight settings, using the SuperCI and then NR algorithm for orbital optimization, and the default CSFCI for the CI step. A segmented all-electron relativistically contracted (SARC) basis set is used for the metal center^78^ and relativistically recontracted Karlsruhe basis sets (DEF2-TZVPP) are used for the other elements^79^. The calculation is sped up by using the RIJK approximation in conjunction with ‘TrafoStep RI’ together with the appropriate “/JK” auxiliary basis sets^80^. The autoaux feature is used to generate the “/JK” auxiliary basis set for the metal center^81^. Overall, since the metal-based orbitals of the active space are mostly non-bonding, and the multi-reference character of the ground state wave function stems from distributing the unpaired electrons mostly among these non-bonding orbitals, it is expected that the multireference character of the wave function will not modify the bonding picture obtained from the AdNDP analysis based on DFT calculations for the investigated systems. For more details, please see CASSCF calculations section of the Supplementary Discussion.

## Supplementary information

Supplementary Information

## Data Availability

The data that support the findings of this study are available from the corresponding author upon reasonable request.
