# Supplementary material for: δ and φ back-donation in AnIV metallacycles
Source: Nat Commun. 2020 Mar 25;11:1558. doi: 10.1038/s41467-020-15197-w (PMC7096508; doi:10.1038/s41467-020-15197-w)
Supplement: Supplementary file 1 — Supplementary Information [file 41467_2020_15197_MOESM1_ESM.pdf]

Supplementary Information

δ and φ Back-Donation in An^{IV} Metallacycles

Morgan P. Kelley,[‡] Ivan A. Popov,[‡] Julie Jung, Enrique R. Batista* & Ping Yang*

[‡]These authors contributed equally.

Theoretical Division, Los Alamos National Laboratory, Los Alamos, New Mexico, USA, 87545.

erb@lanl.gov, pyang@lanl.gov

**Table of Contents**	Pg	Id
Structural properties of the propene complexes	S3	ST1
Structural properties of the cumulene complexes	S4	ST2
Crystal structure references	S5	
Values of the L–M σ and π donations and M–L δ or φ back-donation in propenes and cumulenes	S5	ST3
$C_2(SiMe_3)_2$-to-M π electron donation in actinide propenes and Group 4 propenes	S5	ST4
Effect of the substitution of the trimethylsilyl groups by H atoms	S5	ST5
ON values of the selected bonds of the U-cumulene complex and its H-substituted analogs	S6	ST6
Evolution of the energy gap (in a.u.) between the singly occupied δ orbital and corresponding unoccupied ligand orbital as a function of the An in propenes and cumulenes	S6	ST7
ON values of the 1c-1e, 5c-1e AdNDP bonds and δ/φ back-donation at ECP60MWB_ANO and ECP60MWB_SEG basis sets	S6	ST8
Calculated IR spectra for $(C_5Me_5)_2Th(\eta^4\text{-}1,2,3,4\text{-}PhC_4Ph)$ and $(C_5Me_5)_2U(\eta^4\text{-}1,2,3,4\text{-}PhC_4Ph)$	S6	SF1
Calculated molecular orbitals for the $(\eta^5\text{-}C_5Me_5)_2U[\eta^4\text{-}C_2(SiMe_3)_2]$ complexes (An = Th-Pu)	S7	SF2
Calculated molecular orbitals for the $(\eta^5\text{-}C_5Me_5)_2U[\eta^4\text{-}C_4(SiMe_3)_2]$ complexes (An = Th-Pu)	S7	SF3
AdNDP σ bonds found in propenes	S8	SF4
NPA charges on the M centers in the propene series	S8	SF5
Composition of the M hybrids in the M–C 2c–2e σ bonds in the propene series	S9	SF6
Composition of the C hybrids in the M–C 2c–2e σ bonds in the propene series	S9	SF7
ON values (in |e|) of the M–C σ and M–C–C π bonds in the propene series	S9	SF8
Involvement of the M center in the π and δ bonding with the $C_2(SiMe_3)_2$ ligand for Pa propene	S10	SF9
Unpaired *f*-electrons on the M centers in the propene series	S11	SF10
Three 6c–2e π bonds forming π aromatic set over each C_p ring in the propene series	S12	SF11
Canonical molecular orbitals for the $(\eta^5\text{-}C_5Me_5)_2U[\eta^4\text{-}C_4(SiMe_3)_2]$ complex	S13	SF12
AdNDP σ bonds found in cumulenes	S14	SF13
AdNDP bonding elements found between the $C_4(SiMe_3)_2$ ligand and the M center in the cumulene series	S15	SF14
NPA charges on the M centers in the cumulene series	S16	SF15
Composition of the M hybrids in the M–C_α 2c–2e σ bonds in the cumulene series	S17	SF16
Composition of the C_α hybrids in the M–C_α 2c–2e σ bonds in the cumulene series	S17	SF17
ON values (in |e|) of the M–C_α σ bonds and M–C–C π bonds in the cumulene series	S17	SF18
Involvement of the M center in the π bonding with the $C_4(SiMe_3)_2$ ligand in the cumulene series	S19	SF19
Unpaired *f*-electrons on the M centers in the cumulene series	S21	SF20
Involvement of the M center in the φ bonding with the $C_4(SiMe_3)_2$ ligand in the cumulene series	S22	SF21
Calculated UV-Visible-NIR electron absorption spectra for $(C_5Me_5)_2An(\eta^4\text{-}1,2,3,4\text{-}PhC_4Ph)$ (An = Pa-Pu), UV-Vis region	S23	SF22
Calculated UV-Visible-NIR electron absorption spectra for $(C_5Me_5)_2An(\eta^4\text{-}1,2,3,4\text{-}PhC_4Ph)$ (An = Pa-Pu), near-IR region	S23	SF23
Molecular orbital energy levels in the $(\eta^5\text{-}C_5Me_5)_2An[\eta^4\text{-}C_2(SiMe_3)_2]$ complex using spin-orbit coupling and scalar methods, (An=Th-Pu)	S24-S26	SF24-SF28
Magnitude of the direct L–M σ and π donation and M–L δ or φ back-donation at PBE0	S27	SF29
CASSCF calculations on metallacyclocumulenes	S28-S34	SF30-SF35 ST9-ST13
Supplementary References	S35	

Supplementary Table 1. Structural properties of the propene complexes; C_p groups other than C_5Me_5 are specified where appropriate.

			Distances			Angles	
R	M^{4+}	Source	M-Cp	M-C	C - C	Cp-M-Cp	C- M- C
trimethylsilyl	Ti	This work	2.146(3)	2.122(1)	1.327	138.3	36.4
		Crystal Structure[5]	2.114(6)	2.124(3)	1.309	138.6	35.9
	Zr	This work	2.281(1)	2.241(1)	1.338	139.5	34.8
	Th	This work	2.567(0)	2.396(1)	1.36	140.3	33
	Pa	This work	2.516(4)	2.262(1)	1.387	139.6	35.7
	U	This work	2.48(1)	2.291(3)	1.362	135.8	34.6
		Crystal Structure[7]	2.453(5)	2.33(2)	1.355	138.9	33.3
	Np	This work	2.476(2)	2.322(1)	1.339	137.5	33.5
	Pu	This work	2.477(1)	2.341(1)	1.334	136.3	33.1
phenyl	Ti	This work	2.1375(5)	2.094(0)	1.325	141	36.9
		Crystal Structure[8]	2.109(1)	2.101(3)	1.307	140.7	36.2
	Zr	This work	2.275(0)	2.220(1)	1.338	141.7	35.1
	Th	This work	2.559(0)	2.383(0)	1.362	143.3	33.2
		Crystal Structure[6] $C_5(t\text{-butyl})_2$	2.592(1)	2.395(1)	1.351	138.7	32.6
	Pa	This work	2.495(1)	2.272(1)	1.382	147.7	35.4
	U	This work	2.463(6)	2.284(3)	1.369	147.2	34.9
	Np	This work	2.460(3)	2.289(1)	1.358	144.1	34.5
	Pu	This work	2.4615(5)	2.301(1)	1.350	139.9	34.1

Supplementary Table 2. Structural properties of the cumulene complexes; C_p groups other than C_5Me_5 are specified where appropriate.

			Distances					Angles		
R	M^{4+}	Source	M-Cp	M-C_α	M-C_β	C_α - C_β	C_β - C_β	Cp-M-Cp	C_α- M- C_α	C_α- C_β- C_β
trimethylsilyl	Ti	This work	2.164(0)	2.393(3)	2.223(1)	1.294(1)	1.322	136.1	99.2	153.8(0)
	Zr	This work	2.294(6)	2.444(4)	2.323(1)	1.305(0)	1.321	135.6	96.3	152.8(1)
	Th	This work	2.578(4)	2.586(2)	2.520(1)	1.317(0)	1.320	133.9	90.1	152.7(1)
		Crystal Structure[1]	2.452(3)	2.57(1)	2.505(4)	1.314(0)	1.320	135	90.2	153.15(7)
	Pa	This work	2.53(2)	2.456(0)	2.413(1)	1.331(0)	1.312	132.3	95.0	150.1(0)
	U	This work	2.497(2)	2.480(1)	2.437(0)	1.318(1)	1.318	132.8	93.5	150.6(1)
		Crystal Structure[2]	2.461(6)	2.462(6)	2.439(6)	1.308(3)	1.314	138.1	92.9	148.1(2)
	Np	This work	2.486(0)	2.502(1)	2.428(1)	1.311(0)	1.319	132	93.1	152.0(0)
	Pu	This work	2.507(8)	2.526(4)	2.432(3)	1.304(1)	1.322	127.7	92.4	153.0(0)
phenyl	Ti	This work	2.180(5)	2.26(3)	2.228(0)	1.306(0)	1.311	137.2	104.1	148.6(1)
	Zr	This work	2.297(2)	2.374(2)	2.339(1)	1.316(0)	1.311	137.7	97.4	149.0(1)
		Crystal Structure[3]	2.278(3)	2.35(1)	2.33(1)	1.300(5)	1.327	137.3	97.6	148.3(4)
	Th	This work	2.573(5)	2.552(1)	2.547(1)	1.327(0)	1.311	136.8	90.2	150.2(0)
		Crystal Structure[4] $C_5(t\text{-butyl})_2$	2.532(5)	2.536(6)	2.542(2)	1.323(0)	1.310	137.8	90.3	150.3(0)
	Pa	This work	2.52(1)	2.423(3)	2.439(0)	1.340(1)	1.304	137.0	95.0	147.9(2)
	U	This work	2.489(7)	2.444(2)	2.457(1)	1.330(2)	1.308	136.2	93.8	148.3(2)
		Crystal Structure[2]	2.46(6)	2.462(6)	2.439(6)	1.308(3)	1.314	138.1	92.9	148.1(2)
	Np	This work	2.486(6)	2.460(4)	2.443(0)	1.324(0)	1.309	133.6	93.7	149.6(2)
	Pu	This work	2.480(2)	2.507(2)	2.467(1)	1.312(0)	1.313	136.4	92.0	151.0(2)

Supplementary Table 3. Values of the L–M σ and π donations and M–L δ or φ back-donation in propenes and cumulenes at PBE and PBE0 level of theory.

Propenes							Cumulenes					
Method	PBE			PBE0			PBE			PBE0		
Type	σ	π	δ	σ	π	δ	σ	π	φ	σ	π	φ
Th	0.369	0.138	N/A	0.355	0.133	N/A	0.432	0.368	N/A	0.432	0.353	N/A
Pa	0.429	0.238	0.140	0.391	0.212	0.071	0.536	0.565	0.270	0.519	0.499	0.091
U	0.483	0.216	0.038	0.456	0.196	0.009	0.540	0.576	0.076	0.536	0.507	0.013
Np	0.469	0.186	0.022	0.437	0.157	0.007	0.471	0.574	0.022	0.481	0.520	0.003
Pu	0.440	0.167	0.014	0.409	0.128	0.005	0.403	0.547	0.016	0.395	0.504	0.002

Supplementary Table 4. $C_2(SiMe_3)_2$-to-M π electron donation (in |e|) in actinide propenes and Group 4 propenes at PBE.

Th	Pa	U	Np	Pu	Ti	Zr
0.14	0.24	0.22	0.19	0.17	0.07	0.09

Supplementary Table 5. Effect of the substitution of the trimethylsilyl groups by H atoms on the M–C bond distances in the cumulene series.

An	Original complexes		H-substituted complexes	
	M–C_α, Å	M–C_β, Å	M–C_α, Å	M–C_β, Å
Th	2.586	2.520	2.519	2.539
Pa	2.456	2.412	2.396	2.445
U	2.480	2.437	2.418	2.460
Np	2.502	2.428	2.426	2.449
Pu	2.523	2.432	2.437	2.452

Supplementary Table 6. The occupation number (ON) values (in |e|) of the selected bonds of the U-cumulene complex (A) and its H-substituted analogs before (B) and after (C) the geometry optimization.

Bonds	A	B	C
2c–2e C_α–C_β π bonds //3c–2e U–C_α–C_β π bonds	1.69//1.87	1.73//1.89	1.72//1.89
2c–2e C_β–C_β π bonds//3c–2e U–C_β–C_β σ bonds	1.68//1.87	1.69//1.91	1.69//1.90
2c–2e M–C_α σ bonds//3c–2e U–C_α–C_β σ bonds	1.68//1.95	1.77//1.97	1.77//1.97
Unpaired f-electron//5c–1e φ bond	0.90//0.97	0.92//0.98	0.91//0.98
C_α–Si (C_α–H) σ bonds	1.94	1.97	1.96

Supplementary Table 7. Evolution of the energy gap (in a.u.) between the singly occupied δ/φ orbital and corresponding unoccupied ligand orbital as a function of the An in propenes and cumulenes.

An	Propenes	Cumulenes
Th	-	-
Pa	0.0683	0.0399
U	0.0809	0.0511
Np	0.0908	0.0709
Pu	0.1022	0.0725

Supplementary Table 8. ON values of the 1c-1e, 5c-1e AdNDP bonds and δ/φ back-donation in the Pa-propene and Pa-cumulene complexes at ECP60MWB_ANO and ECP60MWB_SEG basis sets.

Complex	Pa-propene			Pa-cumulene		
Bonding elements	1c-1e	5c-1e	δ bonding	1c-1e	5c-1e	φ bonding
ECP60MWB_ANO	0.7886	0.9281	0.1395	0.6750	0.9446	0.2697
ECP60MWB_SEG	0.7890	0.9280	0.1390	0.6765	0.9445	0.2680

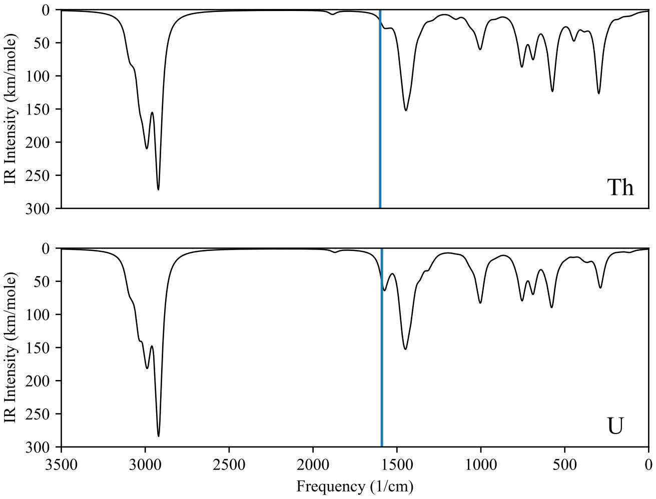

Supplementary Figure 1. Calculated IR spectra for $(C_5Me_5)_2Th(\eta^4\text{-}1,2,3,4\text{-}PhC_4Ph)$ and $(C_5Me_5)_2U(\eta^4\text{-}1,2,3,4\text{-}PhC_4Ph)$. The vertical blue line shows the C_α–C_β stretch reported by Pagano et al.[2] for comparison.

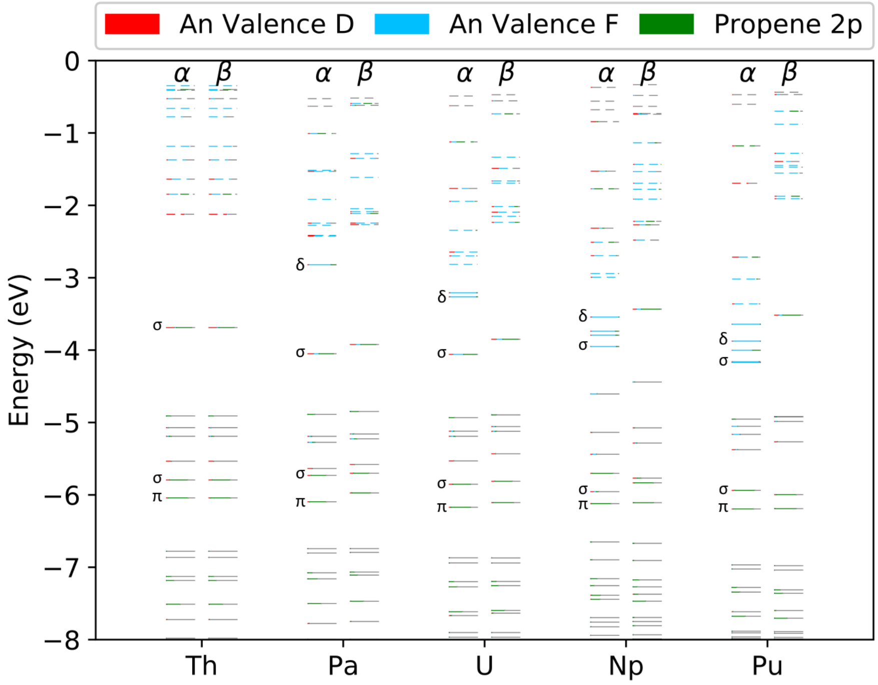

Supplementary Figure 2. Calculated molecular orbitals for the (η^5-C_5Me_5)$_2$U[η^4-$C_2(SiMe_3)_2$] complexes (An = Th, Pa, U, Np, Pu) with An bonding orbitals labelled.

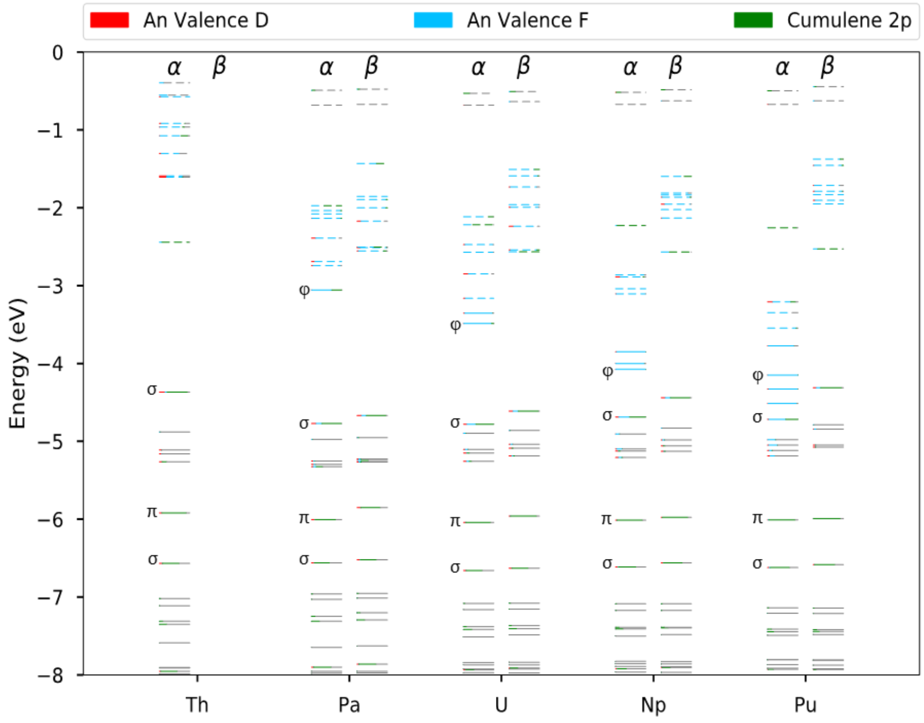

Supplementary Figure 3. Calculated molecular orbitals for the (η^5-C_5Me_5)$_2$U[η^4-$C_4(SiMe_3)_2$] complexes (An = Th, Pa, U, Np, Pu) with An bonding orbitals labelled.

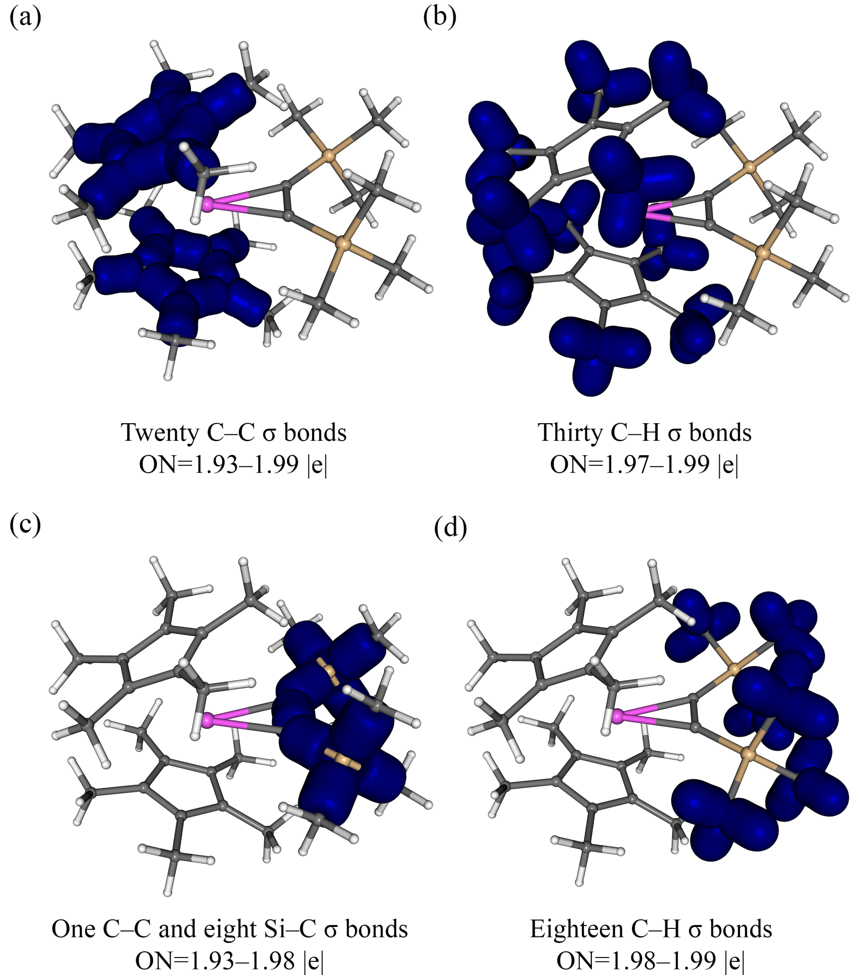

Supplementary Figure 4. AdNDP σ bonds found over the (a, b) $(C_5Me_5)_2$ fragments and (c, d) the $C_2(SiMe_3)_2$ fragments: (a) Five C–C σ bonds found within each C_p ring, five C–C σ bonds connecting the C_p ring and the methyl groups; (b) Thirty C–H σ bonds of the methyl groups connected to C_p rings; (c) One C–C and eight Si–C σ bonds; (d) Eighteen C–H σ bonds. All the bonds are shown as superimposed onto the single molecular framework.

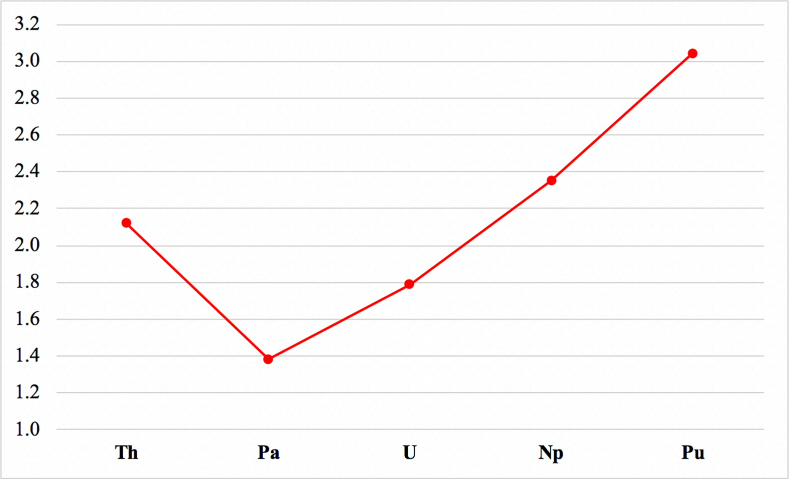

Supplementary Figure 5. NPA charges on the M centers in the propene series.

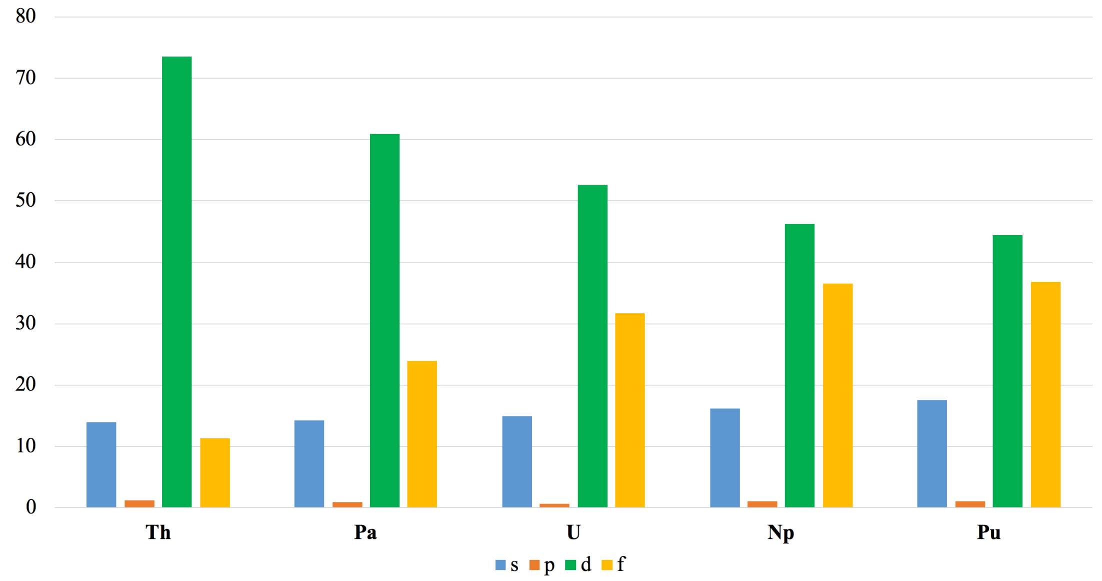

Supplementary Figure 6. Composition (%) of the M hybrids in the M–C 2c–2e σ bonds in the propene series.

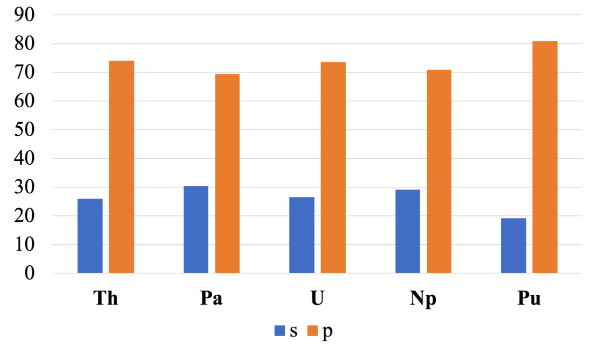

Supplementary Figure 7. Composition (%) of the C hybrids in the M–C 2c–2e σ bonds in the propene series.

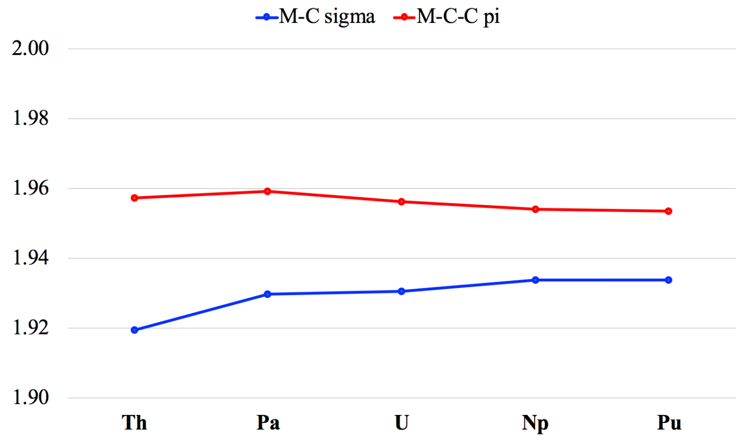

Supplementary Figure 8. ON values (in |e|) of the M–C σ and M–C–C π bonds in the propene series.

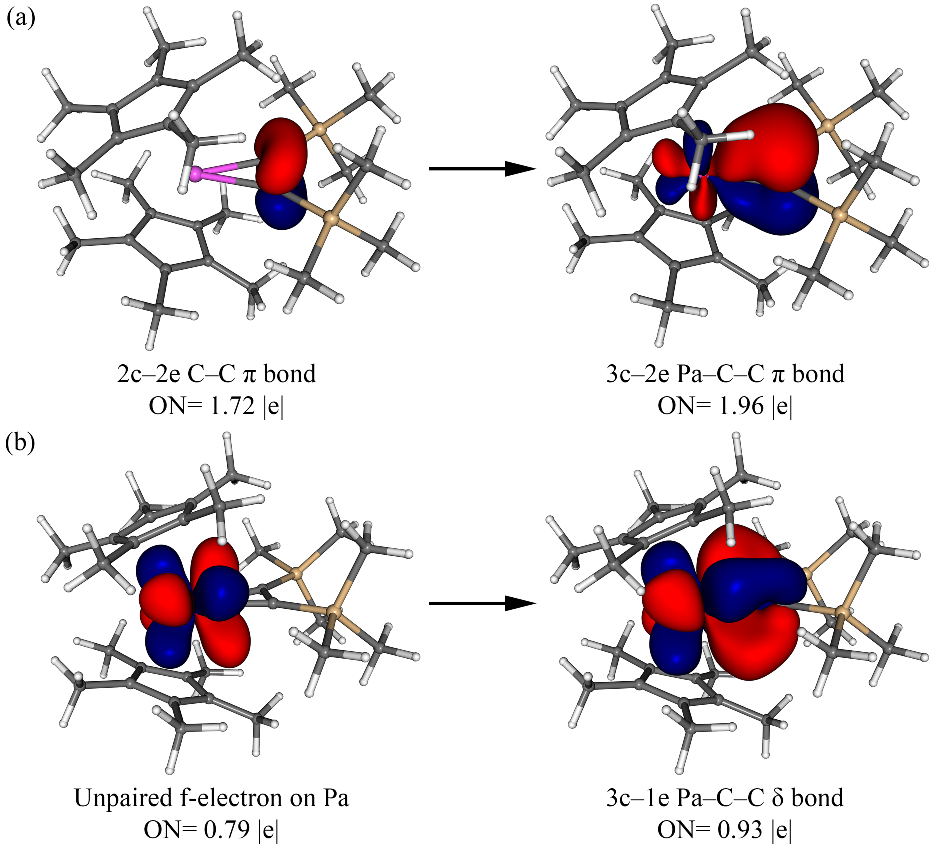

Supplementary Figure 9. Involvement of the M center in the π (a) and δ (b) bonding with the $C_2(SiMe_3)_2$ ligand in the propene series in the example of Pa complex.

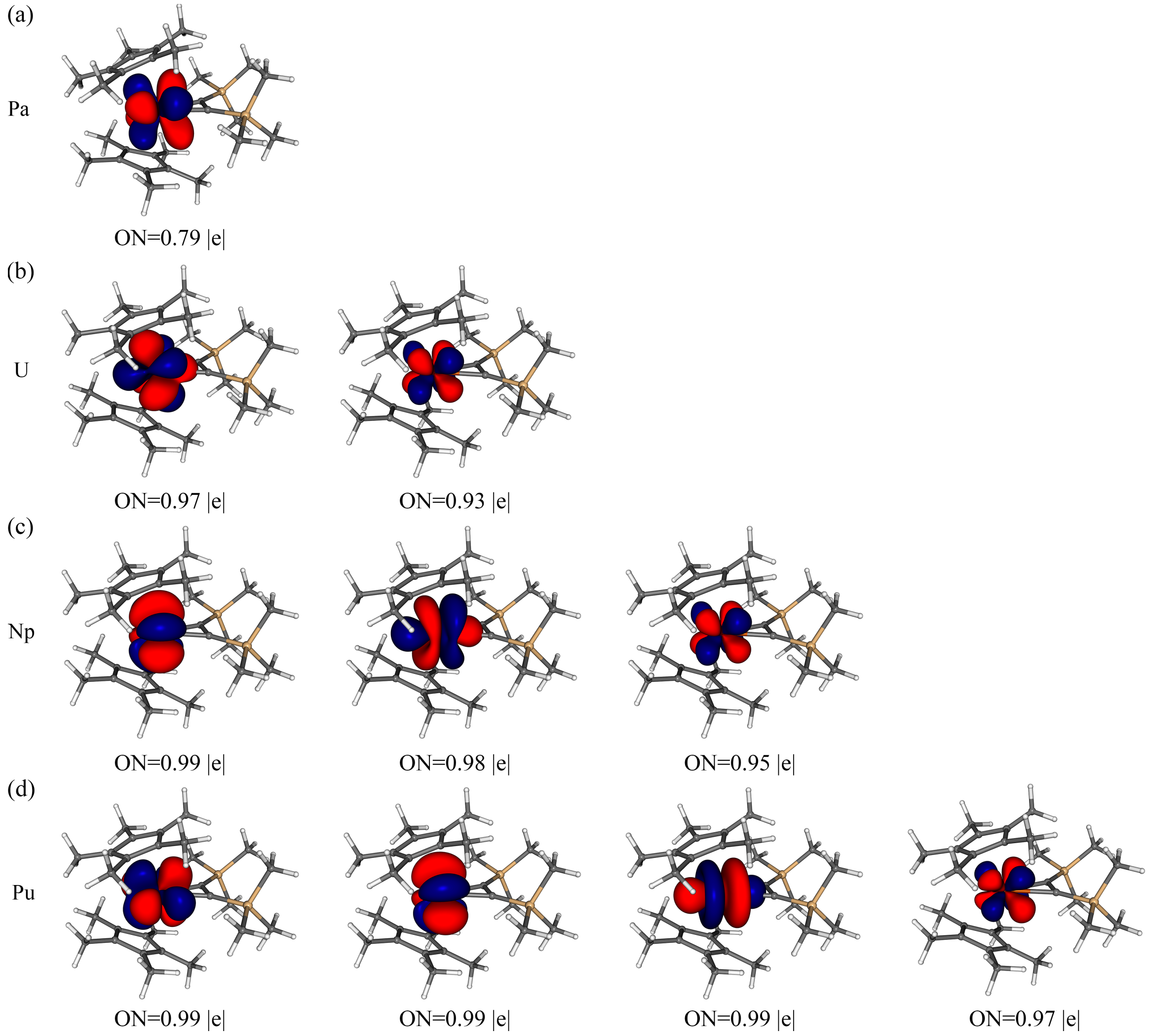

Supplementary Figure 10. Unpaired *f*-electrons (1c-1e) on the M centers in the propene series. (a) One *f*-electron of Pa. (b) Two *f*-electrons of U. (c) Three *f*-electrons of Np. (d) Four *f*-electrons of Pu.

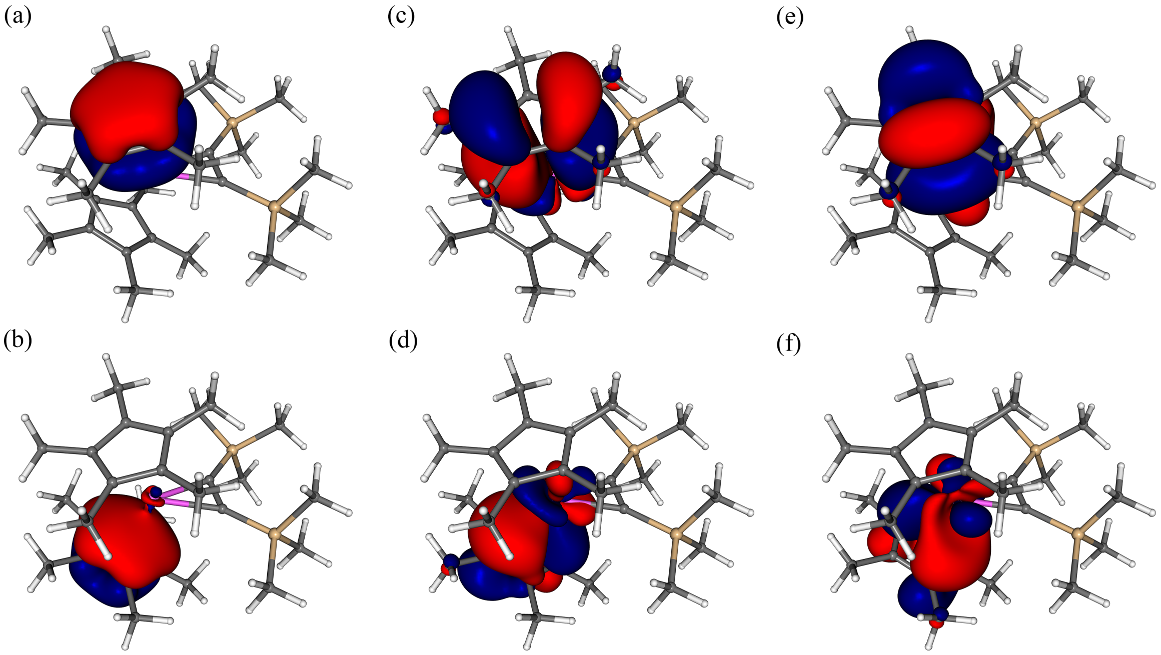

Six 6c–2e π bonds
ON=1.93–1.98 |e|

Supplementary Figure 11. Six 6c–2e π bonds forming π aromatic sets over C_p rings in the propene series. The bonds are mainly composed by the p_z-orbitals of the five C atoms constituting the C_p ring and the *df*-hybridized orbitals of the An atom. While the contribution of the latter into the fully bonding ones (panel a and b) is minor (1–3%), it is appreciably higher (5–12%) in the case of the bonds featuring one nodal plane perpendicular to the C_p ring (panels c, d, e, and f). Similar six 6c–2e π bonds are found in the cumulene series, where the M contribution into these bonds is assessed to be 2–4% and 6–14%, respectively.

Supplementary Discussion

Chemical bonding in metallacyclocumulenes

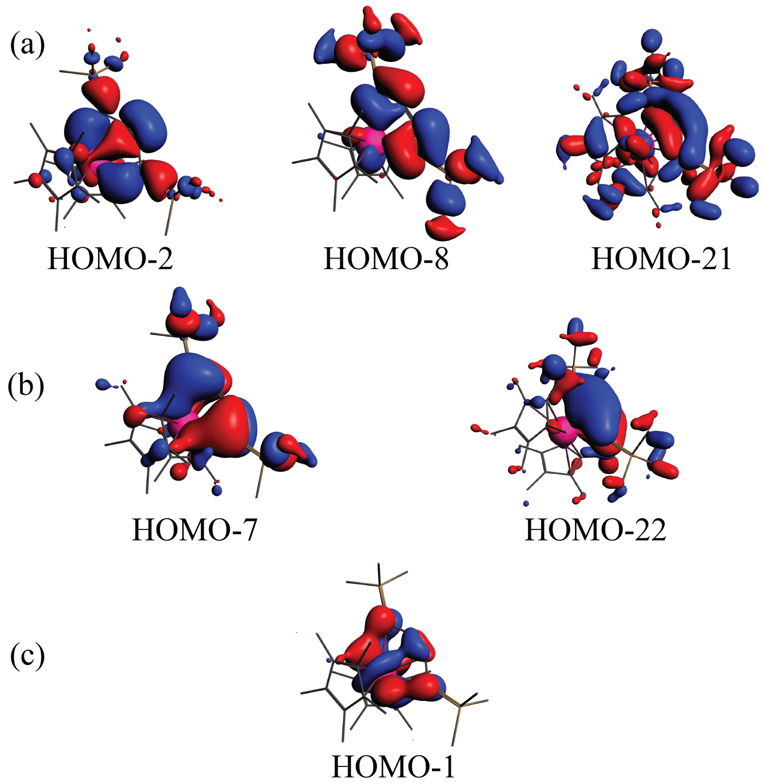

Supplementary Figure 12. Selected canonical molecular orbitals for the (η^5-C_5Me_5)$_2$U[η^4-$C_4(SiMe_3)_2$] complex attributed to (a) M–C σ bonds, (b) π bonds, and (c) the φ bond. ISO = 0.025.

Per AdNDP, the following chemical bonding elements have been recovered out of the totally delocalized CMOs as outlined below.

***σ* bonding**

There are 50 covalent 2c–2e *σ* bonds that constitute the framework of the C_5Me_5 units (20 C–C and 30 C–H *σ* bonds).

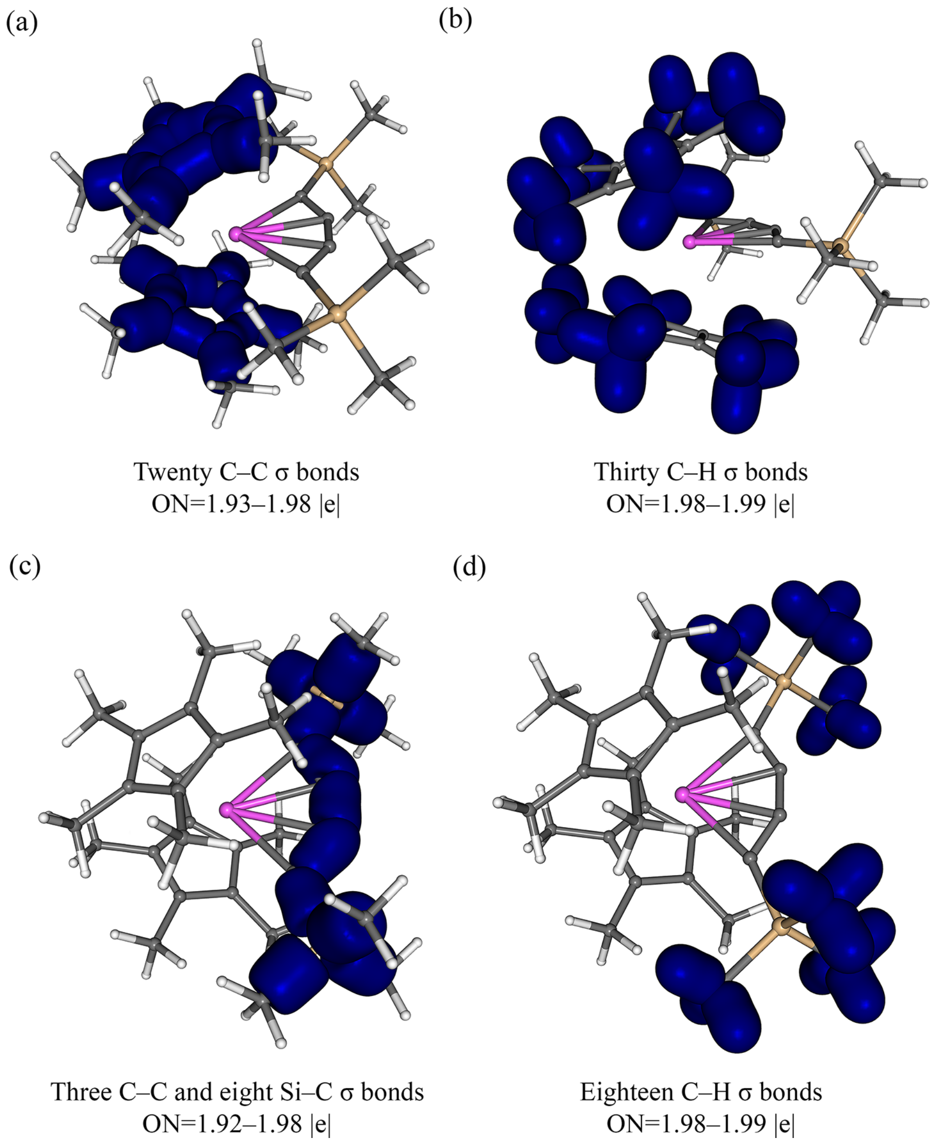

Supplementary Figure 13. AdNDP *σ* bonds found over the (a, b) $(C_5Me_5)_2$ fragments and (c, d) the $C_4(SiMe_3)_2$ fragments: (a) Five C–C *σ* bonds found within each C_p ring, five C–C *σ* bonds connecting the C_p ring and the methyl groups; (b) Thirty C–H *σ* bonds of the methyl groups connected to C_p rings; (c) Three C–C and eight Si–C *σ* bonds; (d) Eighteen C–H *σ* bonds. All the bonds are shown as superimposed onto the single molecular framework.

As seen from Supplementary Fig. 13, 29 2c–2e σ bonds are responsible for the σ bonding within the $C_4(SiMe_3)_2$ ligand (3 C–C, 8 Si–C, and 18 C–H σ bonds). Two M–C_α σ bonds connect the $[MC_5Me_5]^{2+}$ moiety with the cumulene ligand (Supplementary Fig. 14a).

(a)

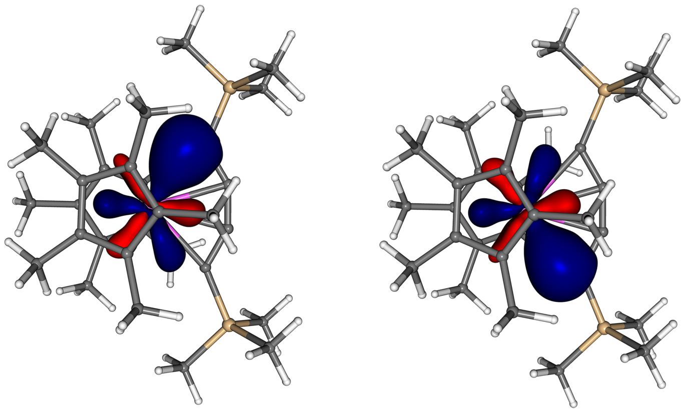

two 2c–2e Pa–C_α σ bonds
ON= 1.68 |e|

(b)

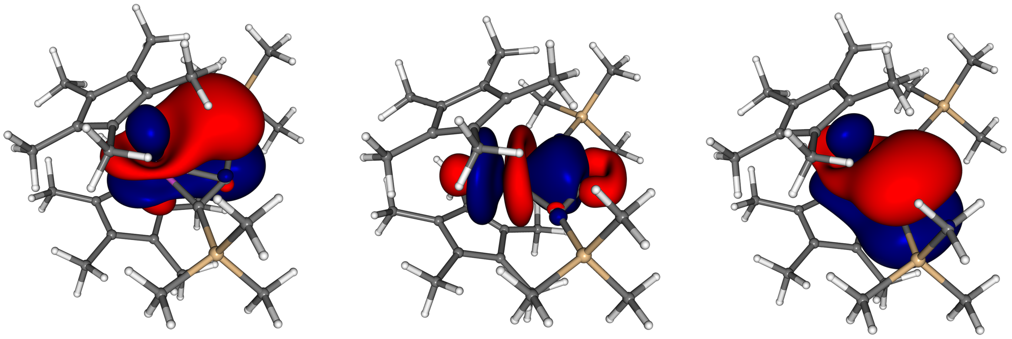

3c–2e Pa–C_α–C_β π bond
ON= 1.86 |e|

3c–2e Pa–C_β–C_β σ bond
ON= 1.89 |e|

3c–2e Pa–C_α–C_β π bond
ON= 1.86 |e|

(c)

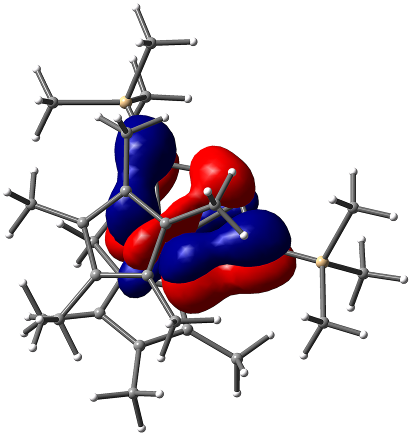

5c–1e Pa–C_α–C_β–C_β–C_α φ bond
ON= 0.94 |e|

Supplementary Figure 14. AdNDP bonding elements found between the $C_4(SiMe_3)_2$ ligand and the M center in the example of Pa-cumulene complex: (a) Two direct Pa–C_α σ bonds; (b) Two peripheral Pa–C_α–C_β π bonds and one central Pa–C_β–C_β σ bond (this bond is considered as σ with respect to the metal center, but π with respect to the C_β–C_β bond); (c) Singly occupied five-center Pa–C_α–C_β–C_β–C_α φ bond.

From one side, the lower ON values of the M–C_α σ bonds in cumulenes (1.65–1.69 |e|) compared to the corresponding values in propenes (1.92–1.93 |e|) explain the elongation of the M–C bonds in the cumulene *vs.* the propene series (2.46–2.59 Å *vs.* 2.26–2.40 Å, respectively). From the other side, the lower ON values of these σ bonds in cumulenes hint at the electron delocalization within this fragment. As a matter of fact, these direct M–C_α σ bonds can also be viewed as two 3c–2e σ bonds involving an additional C_β atom with slightly higher ON values (1.94–1.95 |e|). Contribution of the C_β atoms to these bonds is assessed to be 13.2–16.0%. This finding suggests that the distance between the metal center and the $C_4(SiMe_3)_2$ ligand is mainly controlled by the M–C_α interaction, raising the question of why the C_α atoms – which contribute more electron density to the M–C σ bonds – are located further away than the C_β atoms. To answer this question, we performed additional calculations for model complexes with less bulky groups attached to the C_α atoms. When the bulky trimethylsilyl groups bound to the C_α atoms are substituted for compact hydrogen atoms, the M–C_α bonds become shorter than M–C_β bonds, while the ON values of the corresponding bonds stay nearly the same (Supplementary Tables 5, 6). This suggests that steric effects are largely responsible for keeping the M–C_α bonds longer than M–C_β bonds, while the electronic effects are minor.

The trend in the NPA charges on the metal center is similar to the propene series, with Th (+1.87) possessing larger charge than U (+1.59) (Supplementary Fig. 15). It shows an approximately 0.7 drop in charges between Th (+1.87) and Pa (+1.17) followed by a consistent increase to Pu (+2.85). Similar to propenes, the NPA charge on the metal center of the Pa complex suggests that its reactivity towards alkynes should be comparable to that of the recently synthesized $(\eta^5\text{-}C_5Me_5)_2U[\eta^4\text{-}C_4(SiMe_3)_2]$ as well as Group 4 cumulenes, whereas the reactivity of the corresponding Np and Pu complexes should be similar to the Th complex. As one would expect, the NPA charges of the corresponding Ti and Zr complexes have quite low values (+1.40 and +1.24, respectively), comparable to those of U (+1.59) and Pa (+1.17) complexes.

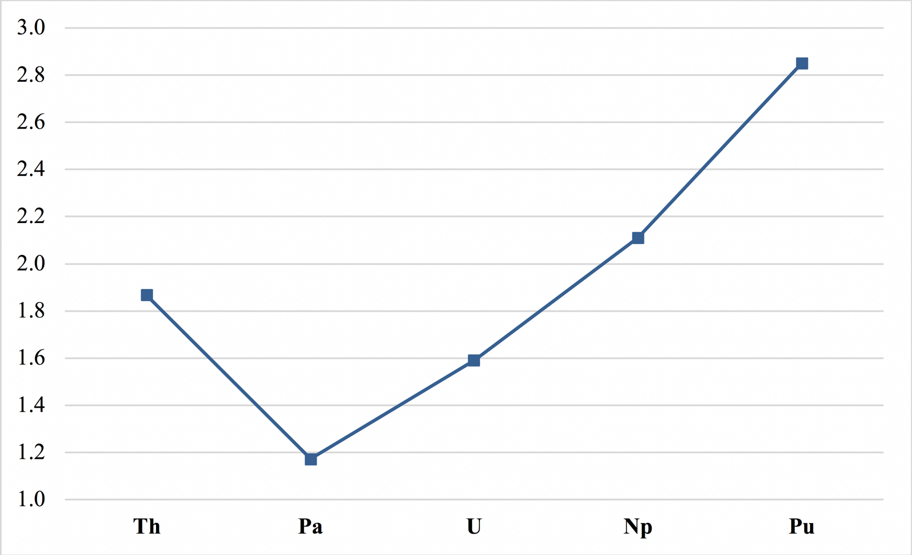

Supplementary Figure 15. NPA charges on the M centers in the cumulene series. NPA charges in corresponding Ti and Zr complexes are +1.40 and +1.24, respectively.

As in the propene series, the cumulene M–C σ bonds are primarily formed by hybrid 6*d*-5*f* An orbitals with C 2*s*-2*p* hybrid orbitals (Supplementary Figs 16, 17), with increasing *f*- and decreasing *d*-characters across the series from Th to Pu. Likewise, the ON values of the M–C σ bonding does not change significantly in the cumulene series (Supplementary Fig. 18).

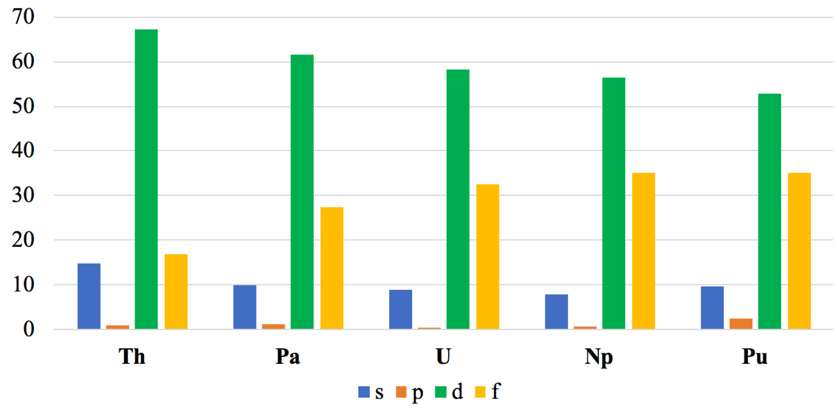

Supplementary Figure 16. Composition (in %) of the M hybrids in the M–C$_\alpha$ 2c–2e σ bonds in the cumulene series.

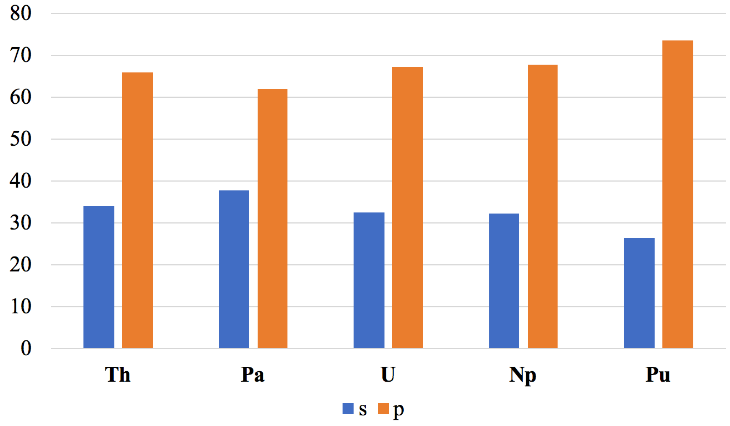

Supplementary Figure 17. Composition (in %) of the C$_\alpha$ hybrids in the M–C$_\alpha$ 2c–2e σ bonds in the cumulene series.

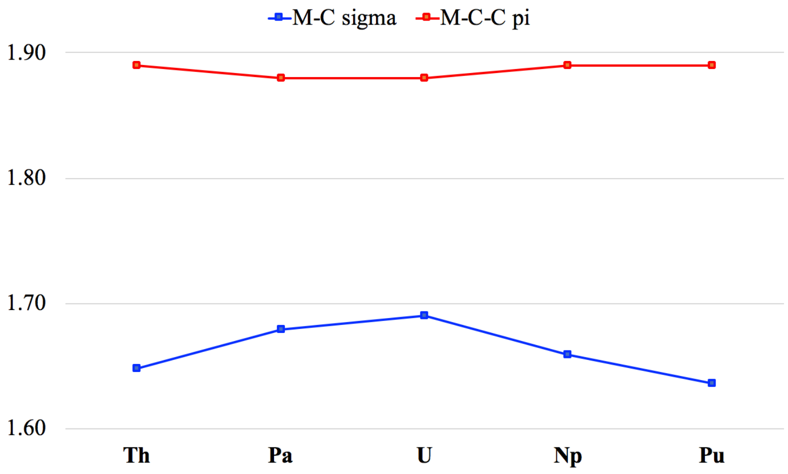

Supplementary Figure 18. ON values (in |e|) of the M–C$_\alpha$ σ bonds and M–C–C π bonds in the cumulene series.

π bonding

The similarity between the cumulenes and propenes extends to their π bonding, though the π interactions are slightly altered in the $(\eta^5\text{-}C_5Me_5)_2An[\eta^4\text{-}C_4(SiMe_3)_2]$ (An=Th–Pu) compounds by the presence of additional C atoms. AdNDP found two types of π bonding interactions between the $(\eta^5\text{-}C_5Me_5)_2An$ and $C_4(SiMe_3)_2$ fragments associated with one central (C_β–C_β) and two peripheral (C_α–C_β) units (Supplementary Fig. 19). Since the M–C_β–C_β bonding is due to the L–M donation of the π density of the C_β–C_β fragment to the *df*-hybrid orbital of the metal center, we call it π interaction. However, the M–C_β–C_β π bonding is indeed formally considered as σ interaction with respect to the metal center. The M–C_β–C_β and M–C_α–C_β π bonds are mutually perpendicular, and are responsible for the double bond character within the four C atoms of the cumulene moiety. These 3c–2e bonds can also be viewed as 2c–2e C–C bonds, though with lower ON values (1.62–1.76 |e|). This hints at π electron delocalization over neighboring atoms (Supplementary Fig. 19). Indeed, upon inclusion of the M atom, the ON values increase to 1.86–1.92 |e|. The L–M π donation is found to be the largest for U (0.58 |e|) and the smallest for Th (0.37 |e|) (Fig. 6b). The larger donation to U compared to the Th center is in agreement with the experimental values of the M–C bonds in the U (avg. 2.45 Å) and Th (avg. 2.54 Å) cumulene complexes. However, similar to the propene series, the combined $\sigma+\pi$ L–M trend does not fully explain the peculiar trend of the M–C distances observed in the An series (Figs. 3a, 6c). Specifically, the Pa-cumulene distances are the shortest in series, while the largest $\sigma+\pi$ L–M donation is found for the U counterpart.

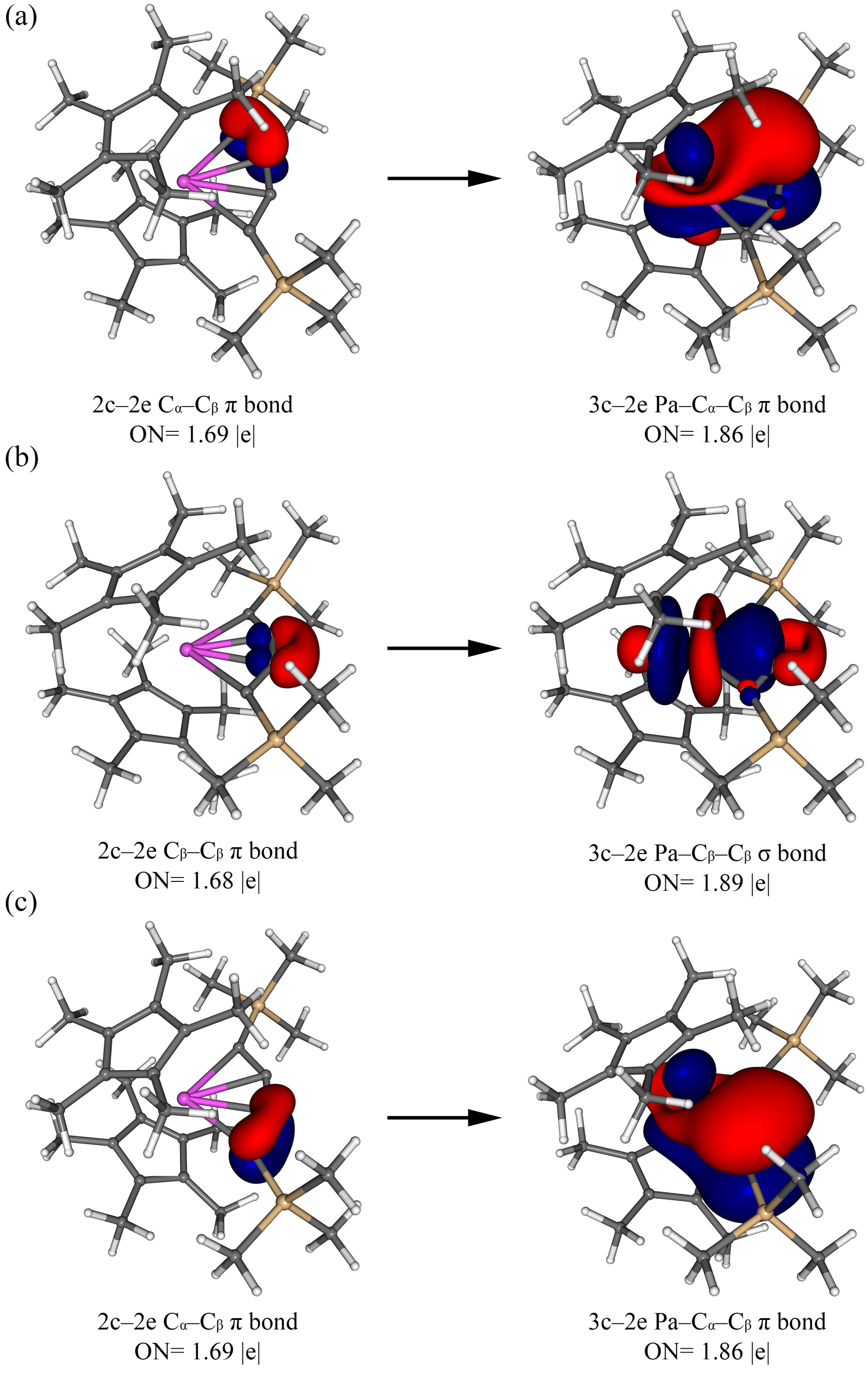

Supplementary Figure 19. Involvement of the M center in the π bonding with the $C_4(SiMe_3)_2$ ligand in the cumulene series in the example of Pa complex. (a) Representation of the 2c–2e C_α–C_β π bond (left) as a 3c–2e Pa–C_α–C_β π bond (right). (b) Representation of the 2c–2e C_β–C_β π bond (left) as a 3c–2e Pa–C_β–C_β π bond (right). The 3c–2e Pa–C_β–C_β bond is considered as σ with respect to the metal center, but π with respect to the C_β–C_β bond. (c) (a) Representation of the 2c–2e C_α–C_β π bond (left) as a 3c–2e Pa–C_α–C_β π bond (right).

φ bonding

While the strength of the interaction between the An atom and the cumulene fragment is primarily dominated by the strength of their σ and π bonding (Fig. 6c), these two interactions alone do not fully explain the peculiar trend of the M–C distances observed in the An series (Fig. 3a). Indeed, excepting Pa, the combined $\sigma+\pi$ L–M interactions can account for the M–C bond length decrease from Th to Pa and follow-up constant increase from Pa to Pu, thus defying the anticipated trend of ionic radii in actinides. However, there is more one interaction, which is found to be the strongest in the case of the Pa complex, *i.e.* M–L φ back-bonding (Fig. 6d) that helps to bring the donation trend in full consistency with the M–C bond trend (Figs. 3a, 6e).

Specifically, one of the unpaired *f*-electrons of the M atom, which has the lowest ON value (Supplementary Fig. 20), participates in a φ bond with the four C atoms of the $C_4(SiMe_3)_2$ fragment. Similar to the Group 4 cumulenes, no φ bonding interaction is observed in the case of the Th complex due to the absence of *f*-electrons. The 5c–1e φ bond is formed due to the promotion of the electron density from the singly occupied $5f_{xz^2}$ orbital of the An atom into the π^* orbital of the four C atoms of the $C_4(SiMe_3)_2$ ligand (Supplementary Fig. 21). It features three nodal planes: one nodal plane going through the plane of the four C atoms in addition to two perpendicular nodal planes, in between the C_α and C_β atoms on both sides of the ligand. This unprecedented 5c–1e φ bond is the first example of the "side-to-side" M–L φ interaction occurring between the 5*f* orbital of the metal and the π^* orbital of the ligand.

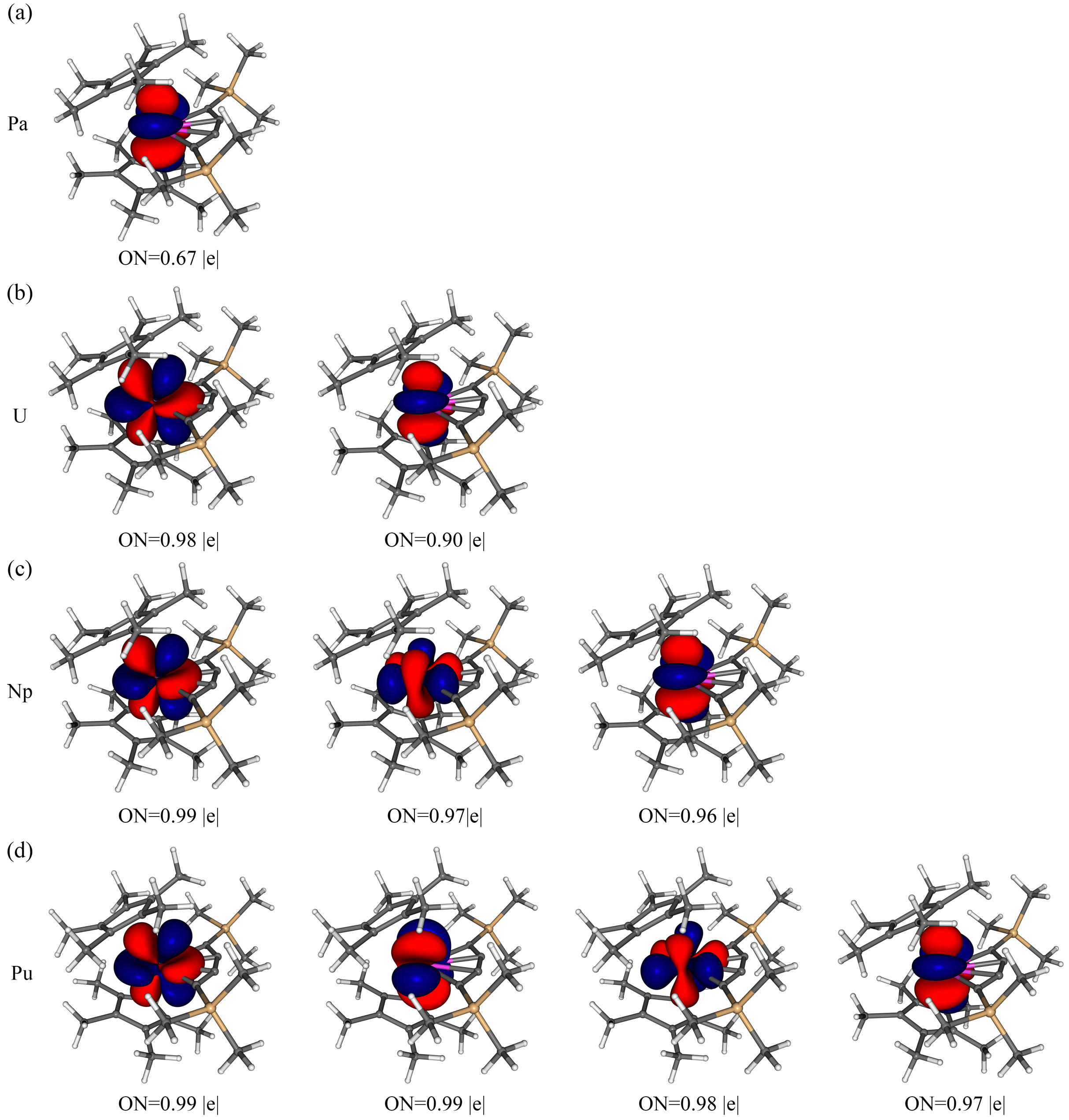

Supplementary Figure 20. Unpaired *f*-electrons (1c-1e) on the M centers in the cumulene series. (a) One *f*-electron of Pa. (b) Two *f*-electrons of U. (c) Three *f*-electrons of Np. (d) Four *f*-electrons of Pu.

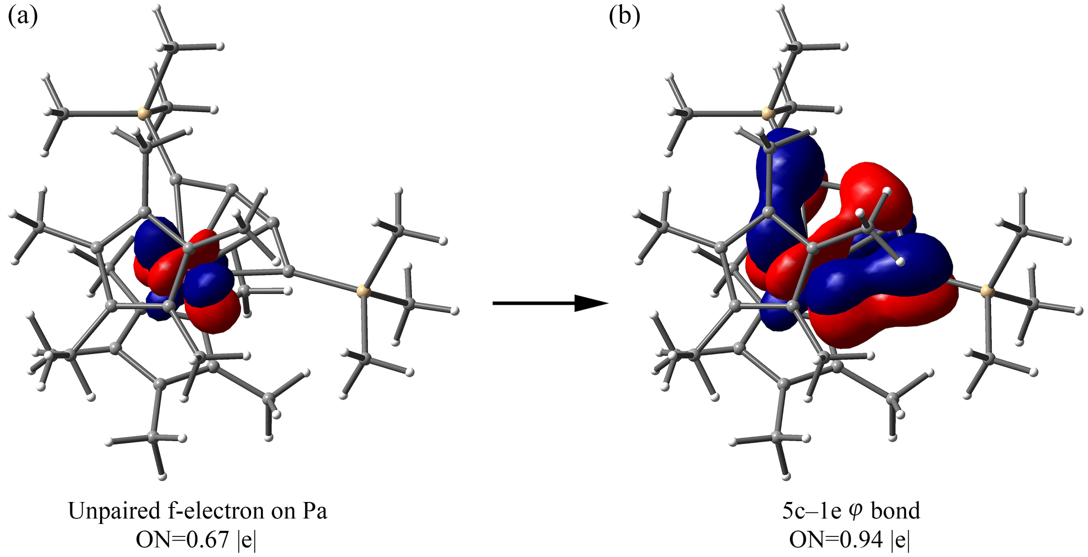

Supplementary Figure 21. Involvement of the M center (1c-1e, f-electron) (a) in the φ bonding with the $C_4(SiMe_3)_2$ ligand (5c-1e M-C-C-C-C) (b) in the cumulene series in the example of Pa complex.

On one hand, the φ back-donation is expected destabilize the C_α–C_β bonds due to their antibonding characters with respect to one another as well as to stabilize C_β–C_β exhibiting bonding interactions. The degree of the φ back-donation within the series (Fig. 6d) is in agreement with the C_α–C_β bond lengths, confirming the longest C_α–C_β bonds in the Pa complex featuring the largest φ back-bonding. In contrast to the C_α–C_β bond length trend, the C_β–C_β bonds stay nearly the same along the series. This in agreement with the fact that the back-donation to the central C_β atoms is significantly smaller (27%) than to the peripheral C_α (73%).

On the other hand, the φ back-donation is expected to stabilize the M–C_α interaction, resulting in shorter M–C_α bonds depending on the degree of such donation. As in propenes, the M–L φ back-bonding is strongest in Pa (0.27 |e|); however, the φ interaction of U (0.08 |e|) cumulene is significantly stronger than that of Np (0.02 |e|) or Pu (0.02 |e|). While the U–cumulene φ back donation is still considered as a minor effect since it does not impact the overall donation trend, it does so in the case of Pa. Specifically, accounting for the M–L φ donation results in the highest overall donation value for Pa instead of U (Fig. 6e), in agreement with the shortest Pa-cumulene distance. Although the φ back-donation is small for Np (0.02 |e|) and Pu (0.02 |e|), it grows to 0.08 |e| in U, and to 0.27 |e| in Pa, for which it is even larger than the direct L–M π donation from any C–C π bonds taken alone (0.18 |e|, 0.19 |e|). Overall, presence of the M–L φ interactions in the cumulenes supports the observed M–C bond length trend, thus confirming its indispensable role in explaining of the peculiar geometrical changes found in the cumulene series, particularly for Pa.

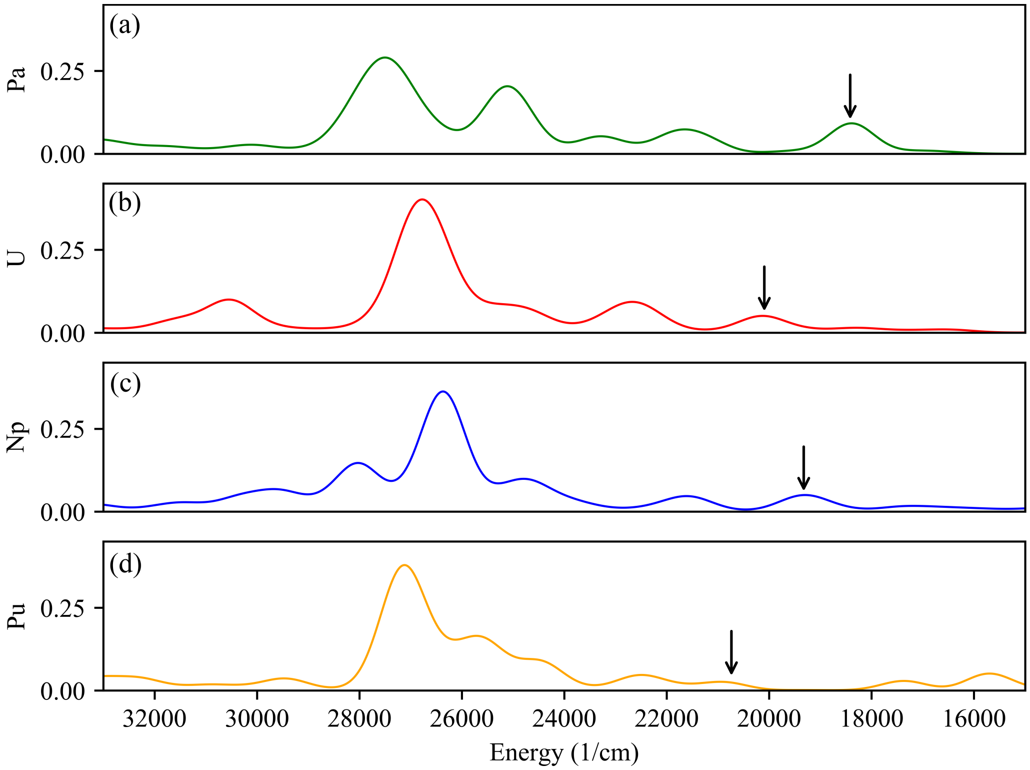

Supplementary Figure 22. Calculated UV-Visible-NIR electron absorption spectra for $(C_5Me_5)_2An(\eta^4$-1,2,3,4-$PhC_4Ph)$ (An = Pa (a), U (b), Np (c), Pu (d)), UV-Vis region. Arrows point to the peaks corresponding to the transitions involving the φ bonding orbital.

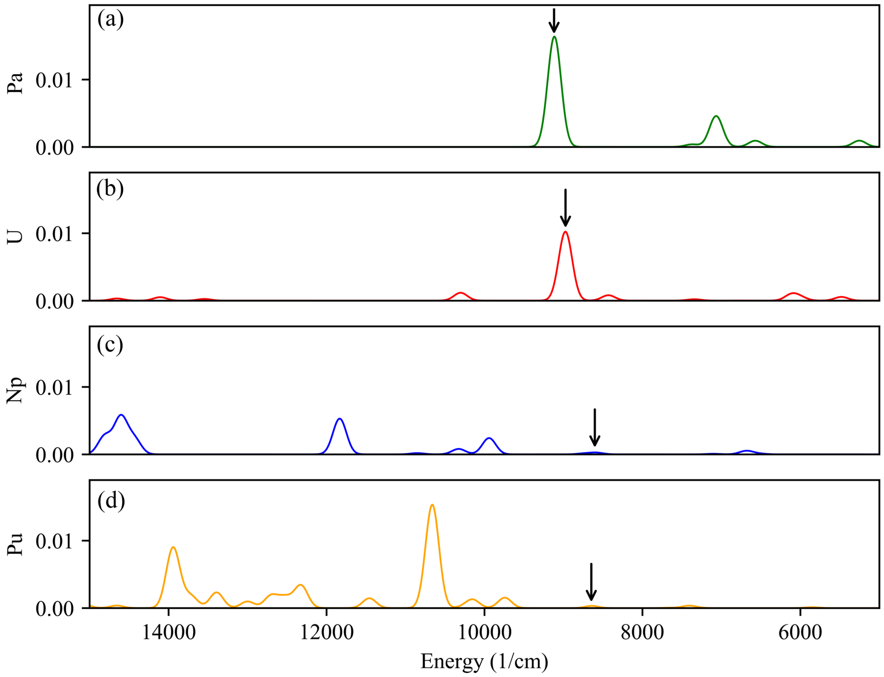

Supplementary Figure 23. Calculated UV-Visible-NIR electron absorption spectra for $(C_5Me_5)_2An(\eta^4$-1,2,3,4-$PhC_4Ph)$ (An = Pa (a), U (b), Np (c), Pu (d)), near-IR region. Plotted separately from UV-Vis region due to much lower intensity. Arrows point to the peaks corresponding to the transitions involving the φ bonding orbital.

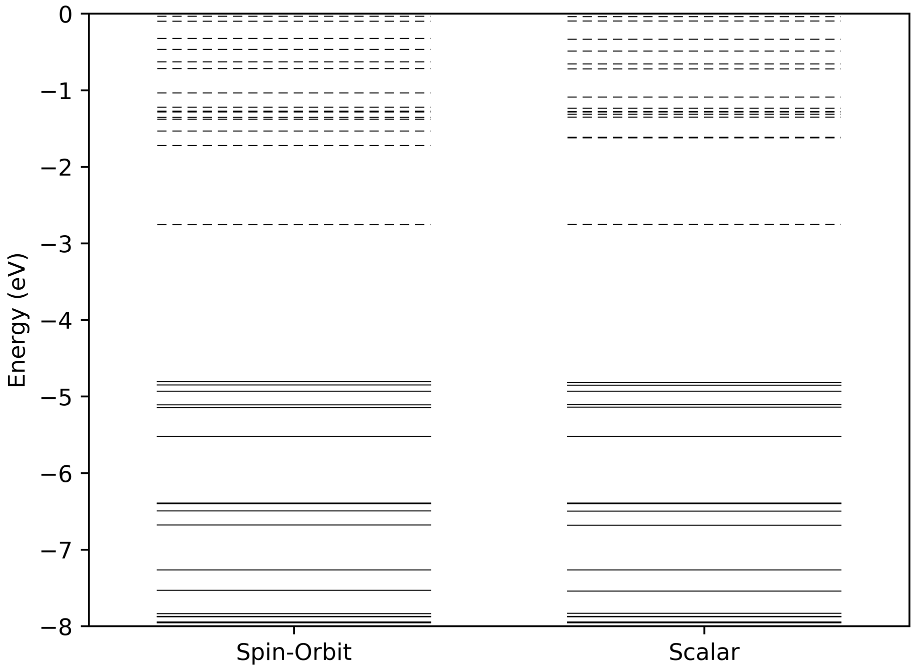

Supplementary Figure 24. Molecular orbital energy levels in the (η^5-C_5Me_5)$_2$Th[η^4-$C_2(SiMe_3)_2$] complex using spin-orbit coupling and scalar methods. Solid lines depict occupied orbitals, dashed lines unoccupied orbitals.

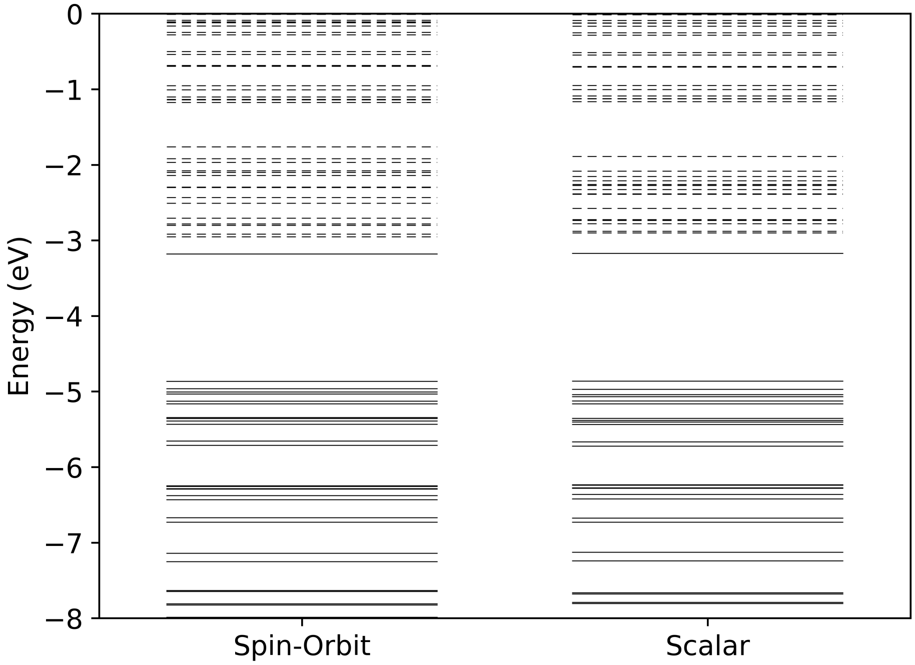

Supplementary Figure 25. Molecular orbital energy levels in the (η^5-C_5Me_5)$_2$Pa[η^4-$C_2(SiMe_3)_2$] complex using spin-orbit coupling and scalar methods. Solid lines depict occupied orbitals, dashed lines unoccupied orbitals.

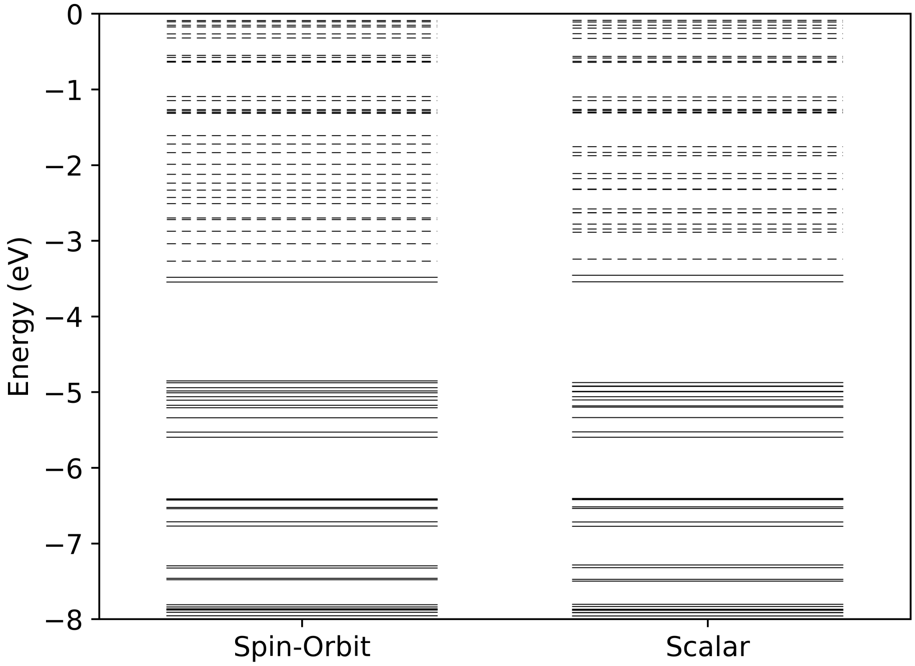

Supplementary Figure 26. Molecular orbital energy levels in the (η^5-$C_5Me_5)_2U[\eta^4$-$C_2(SiMe_3)_2$] complex using spin-orbit coupling and scalar methods. Solid lines depict occupied orbitals, dashed lines unoccupied orbitals.

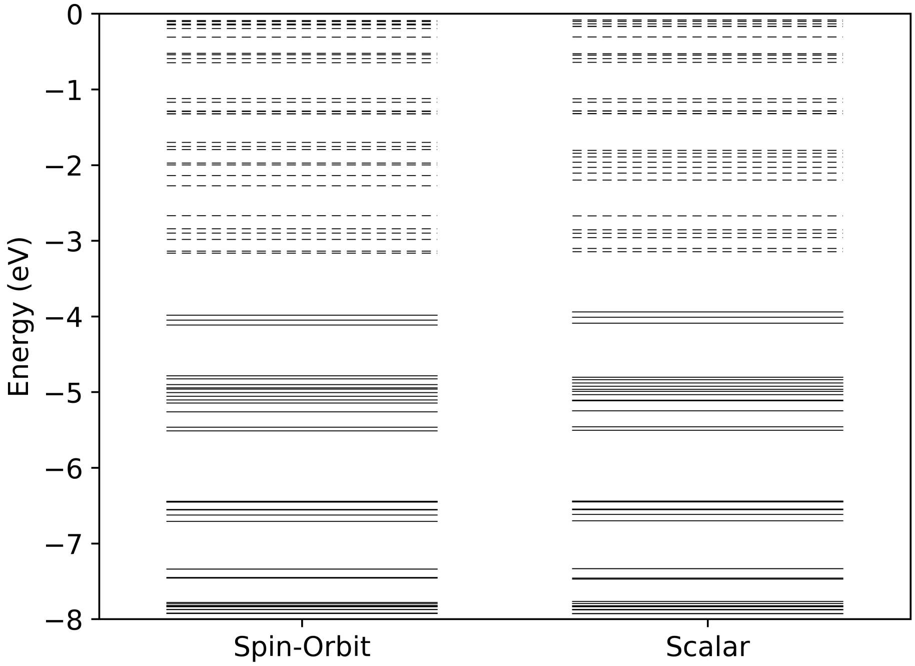

Supplementary Figure 27. Molecular orbital energy levels in the (η^5-$C_5Me_5)_2Np[\eta^4$-$C_2(SiMe_3)_2$] complex using spin-orbit coupling and scalar methods. Solid lines depict occupied orbitals, dashed lines unoccupied orbitals.

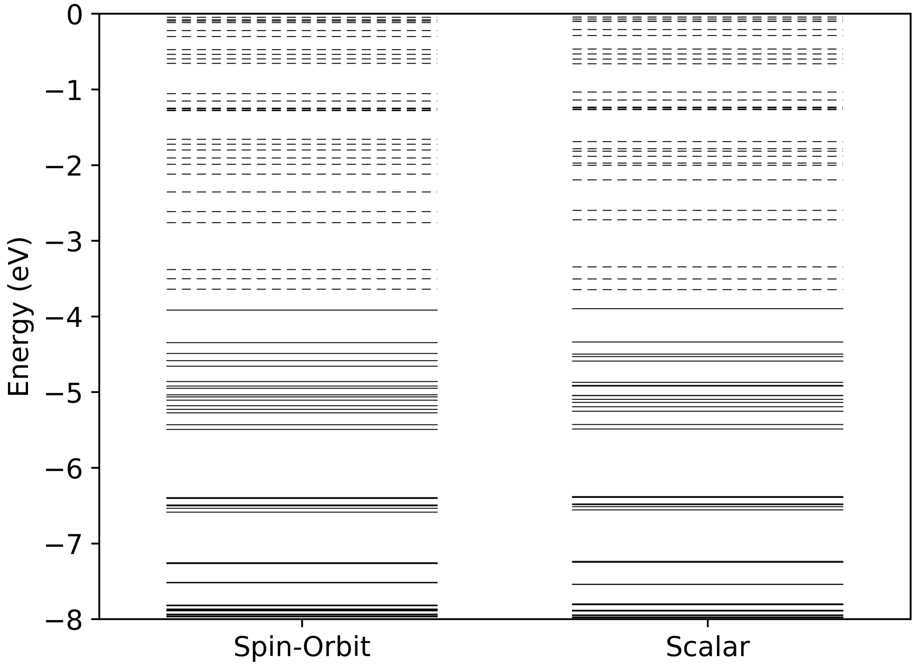

Supplementary Figure 28. Molecular orbital energy levels in the (η^5-C_5Me_5)$_2$Pu[η^4-$C_2(SiMe_3)_2$] complex using spin-orbit coupling and scalar methods. Solid lines depict occupied orbitals, dashed lines unoccupied orbitals.

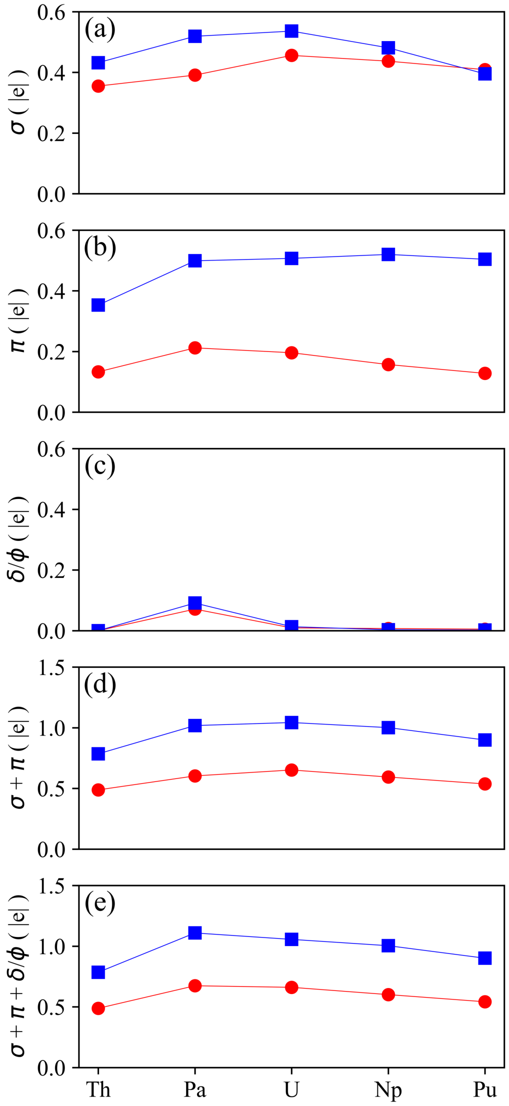

Supplementary Figure 29. Magnitude of the direct L–M σ and π donations and M–L δ or φ back-donation between the $C_2(SiMe_3)_2$ (propene series (δ), red circles) and $C_4(SiMe_3)_2$ (cumulene series (φ), blue squares) ligands and the M center in the Th–Pu series. (a) L–M σ donation. (b) L–M π donation. (c) M–L δ/φ back-donation. (d) L–M (σ+π) donation. (e) Overall (σ+π+δ/φ) donation. Data taken from hybrid PBE0 calculations; compare to Figure 6.

CASSCF calculations

In order to assess the multi-reference character of the electronic wave-function in the Pa, U, Np and Pu derivatives of the cumulene series, CASSCF calculations with an active space containing ligand orbitals of the cumulene ligand were carried out. The calculations confirmed the strong multi-reference character of the wave functions of the ground spin-orbit state for each system. As expected from Hund's rules, the ground state wave function is dominated by determinants of the highest spin state in all cases: doublet for Pa, triplet for U, quartet for Np, and quintet for Pu. Specifically, CASSCF calculations considering the 6 σ- and π-type orbitals that describe the π bonds in the cumulene ligand (3 bonding orbitals + 3 anti-bonding orbitals) were performed. Indeed, these orbitals are more likely to mix with the metal f orbitals than those describing σ bonds within the cumulene ligand.

Since CASSCF calculations rapidly become expensive with increasing active space, not all f orbitals are included in the active space. The number of f orbitals to be included is determined using ab initio ligand field theory (AILFT). Details about this approach can be found in the supplementary information attached to Jung *et al.* Inorg. Chem. 2017, 56, 8802-8816. In a nutshell, in this approach, the CASSCF energies and wave functions of a CAS(n,7) calculation are fitted against a model Hamiltonian that incorporates the effects of electron-electron repulsion, spin-orbit coupling and ligand field interactions. The fit yields a series of parameters for each of the aforementioned interactions. In the case of ligand field interactions, the fit yields the 7x7 matrix of the ligand field Hamiltonian expressed in the basis of the real f-orbitals. The functions that diagonalize this matrix are called ligand field orbitals. They effectively describe how the ligand field interaction splits energetically the seven f orbitals. The shape and relative energy of these orbitals are shown in Supplementary Figure 30 for the cumulene series. The orbitals included in the active space are shown with a black box for each derivative.

The number of computed roots depends on the number of metal orbitals included in the active space, such that only roots contributing to the ground spin-orbit state are computed. Despite the contribution of lower spin states highlighted in the CAS(n,7) calculations, only roots of the highest spin multiplicity are considered for each system. All roots are equally weighted in the state average calculation. Supplementary Table 9 summarizes the computational settings for each system.

Supplementary Table 9. Summary of the CASSCF - extended active space.

	Pa	U	Np	Pu
Configuration	f^1	f^2	f^3	f^4
Active space	CAS(7,9)	CAS(8,9)	CAS(9,12)	CAS(10,13)
Number of f orbitals in the active space	3	3	6	7
Spin multiplicities (S)	1/2	1	3/2	2
Number of roots	3	3	4	2

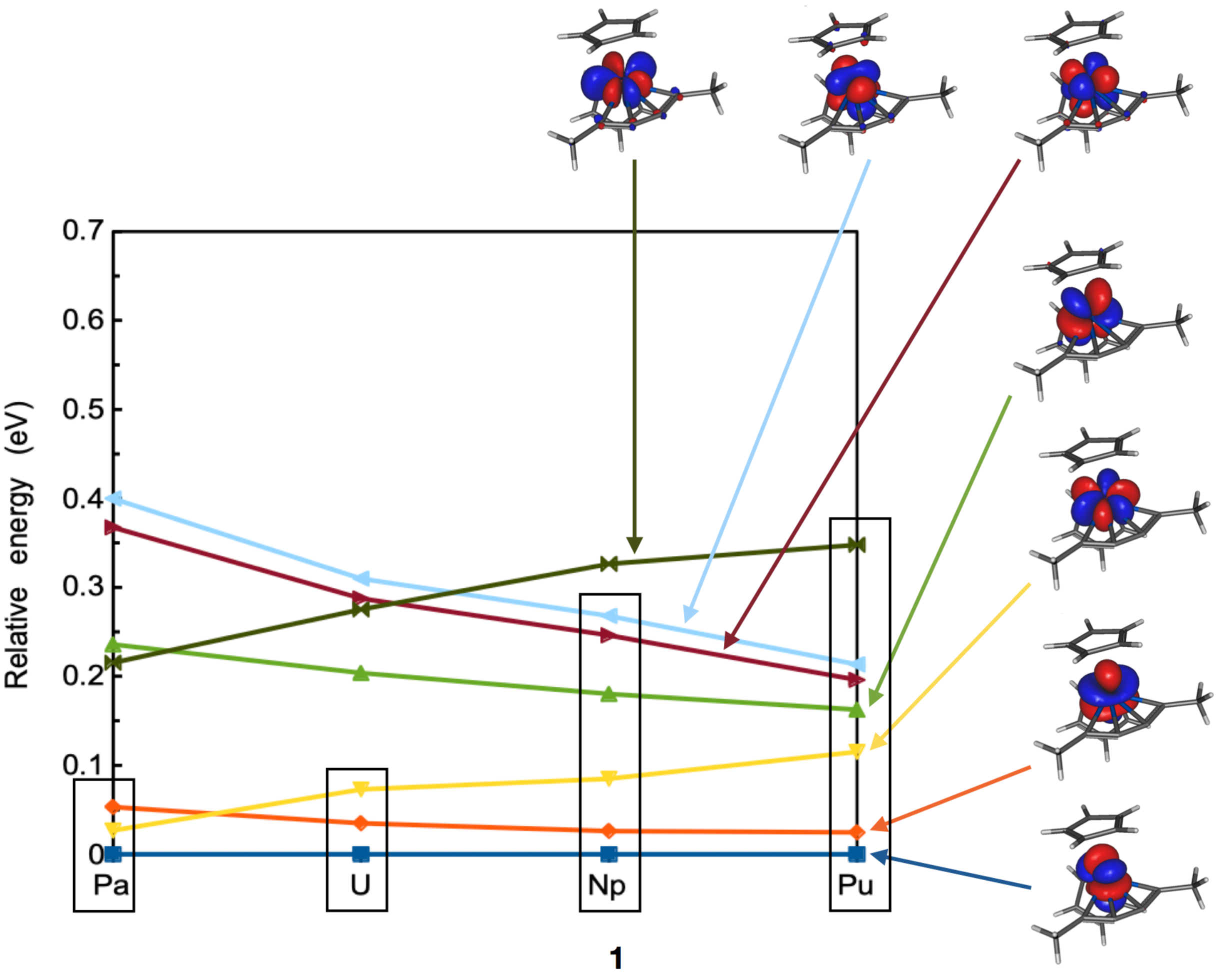

Supplementary Figure 30. Crystal field splitting of the f orbitals. The black box represents the orbitals included in the active space.

The active space orbitals of the extended CAS calculations are shown in Supplementary Figures 31, 32, 33, and 34 for the Pa, U, Np and Pu derivatives, respectively. The associated ground spin orbit state wave functions are shown in Supplementary Tables 10, 11, 12, and 13, respectively.

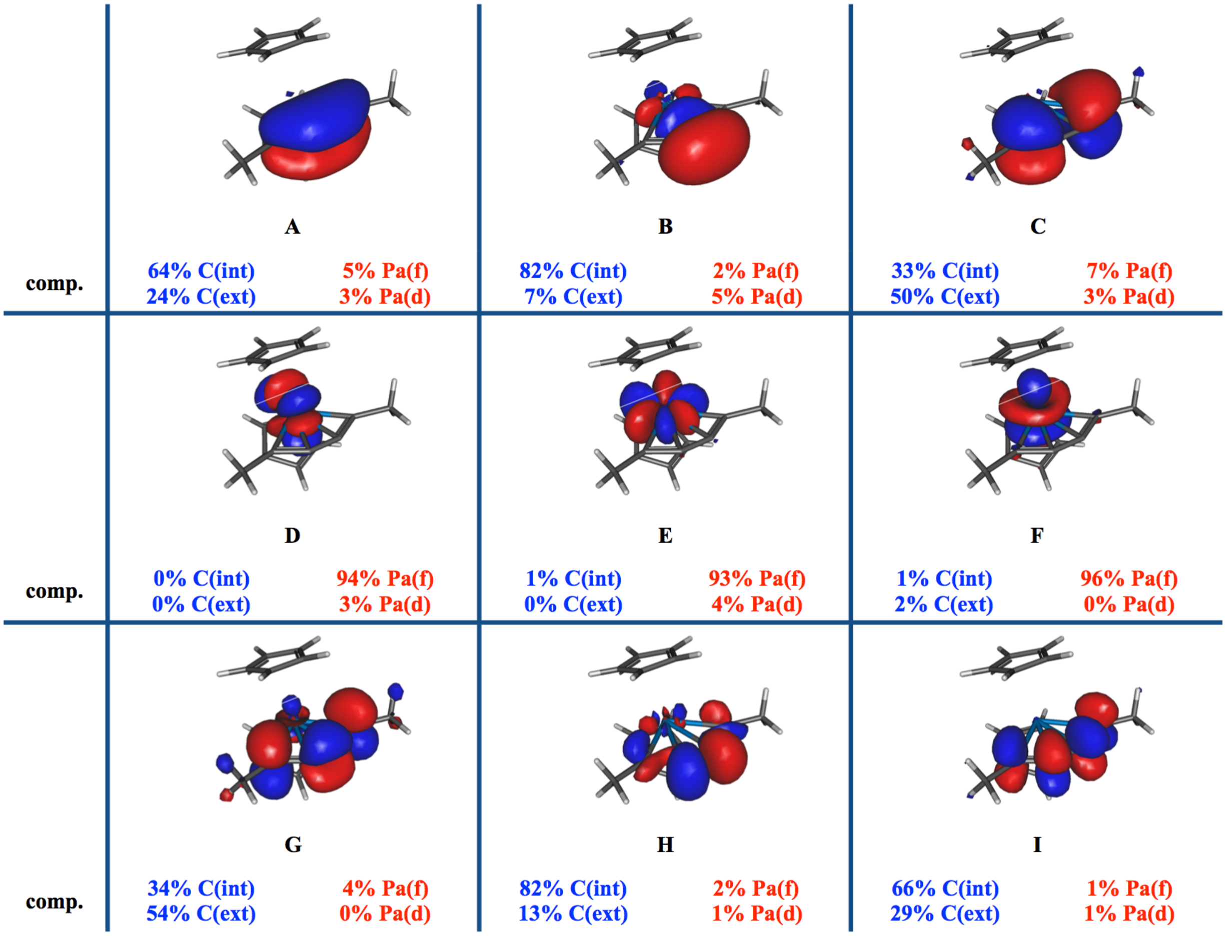

Supplementary Figure 31. Active space MOs for the Pa derivative, with their composition in terms of atomic orbitals (isosurface = 0.03). C(int) corresponds to the internal carbon atoms, while C(ext) corresponds to the peripheral carbon atoms of the cumulene ligand. Orbitals A (G), B (H), and C(I) are the bonding (anti-bonding) orbitals built from the π bond in the cumulene ligand. Orbitals D, E, and F are metal-based orbitals, with dominant f character.

Supplementary Table 10. Electronic wave function of the ground spin-orbit state, in terms of determinants for the Pa derivative. Only determinants contributing by more than 1% are shown. The spin component is neglected. Determinants are written down with respect to the occupation number of the different f orbitals shown in Supplementary Figure 31.

orbitals/ weight	A	B	C	D	E	F	G	H	I
55%	2	2	2	1	0	0	0	0	0
30%	2	2	2	0	1	0	0	0	0
4%	2	2	2	0	0	1	0	0	0

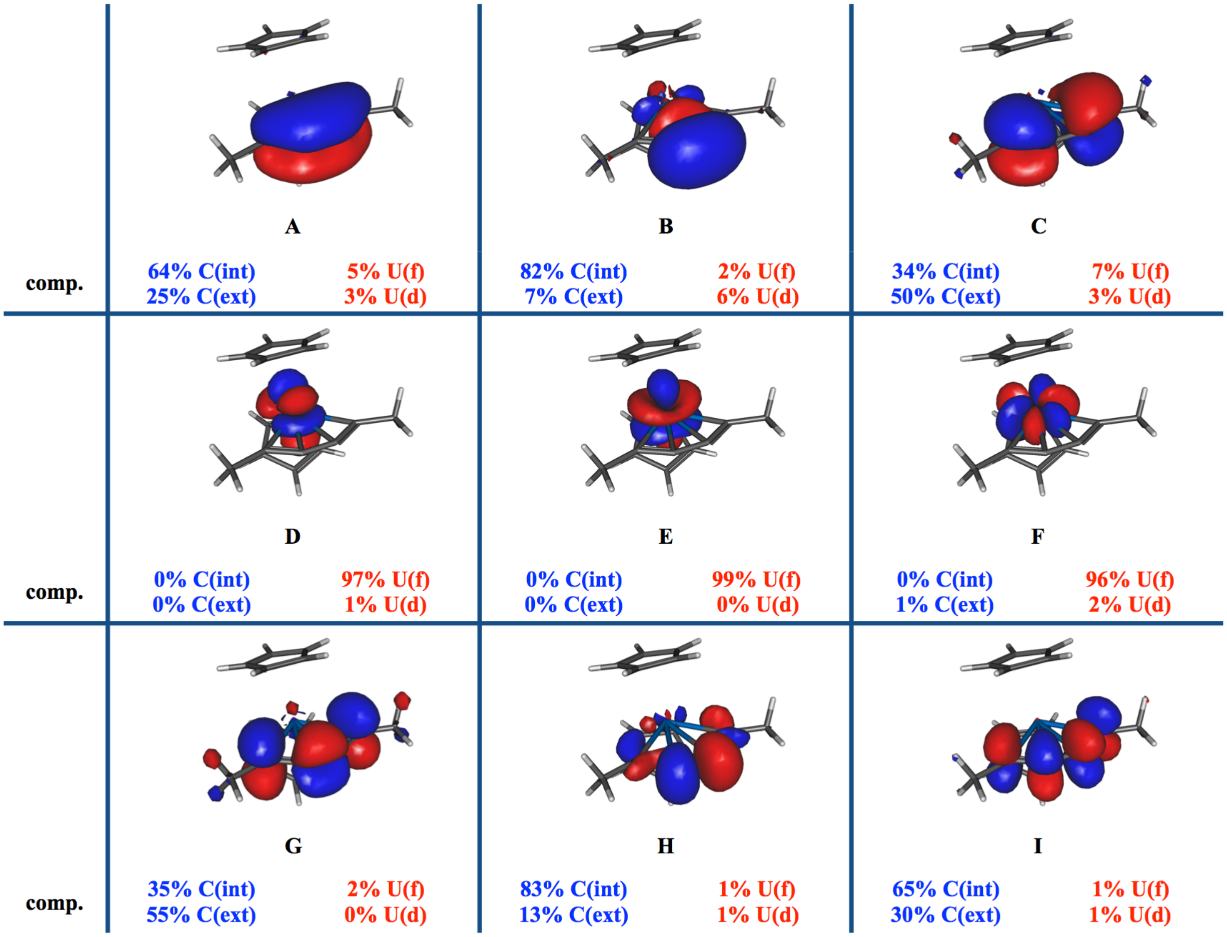

Supplementary Figure 32. Active space MOs for the U derivative, with their composition in terms of atomic orbitals (isosurface = 0.03). C(int) corresponds to the internal carbon atoms, while C(ext) corresponds to the peripheral carbon atoms of the cumulene ligand. Orbitals A (G), B (H), and C(I) are the bonding (anti-bonding) orbitals built from the π bond in the cumulene ligand. Orbitals D, E, and F are metal-based orbitals, with dominant f character.

Supplementary Table 11. Electronic wave function of the ground spin-orbit state, in terms of determinants for the U derivative. Only determinants contributing by more than 1% are shown. The spin component is neglected. Determinants are written down with respect to the occupation number of the different f orbitals shown in Supplementary Figure 32.

orbitals / weight	A	B	C	D	E	F	G	H	I
80%	2	2	2	1	1	0	0	0	0
9%	2	2	2	0	1	1	0	0	0

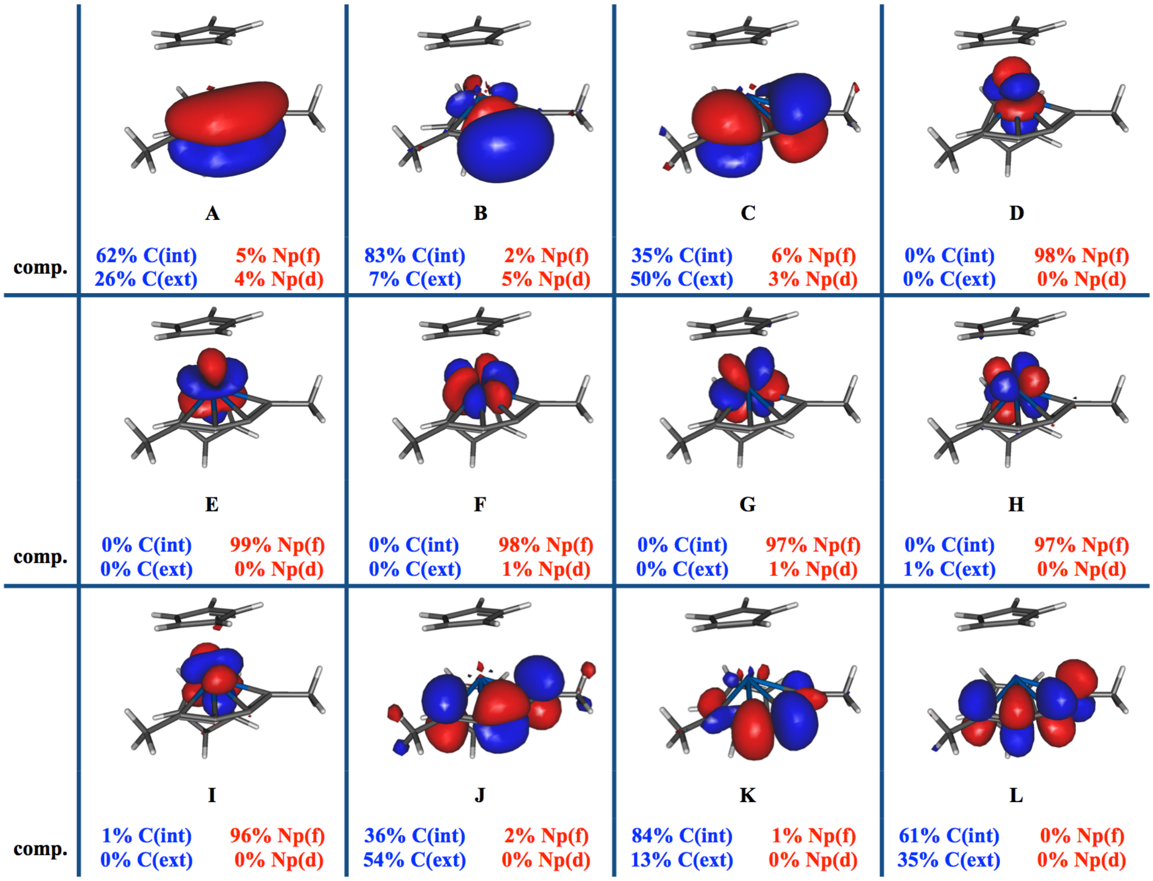

Supplementary Figure 33. Active space MOs for the Np derivative, with their composition in terms of atomic orbitals (isosurface = 0.03). C(int) corresponds to the internal carbon atoms, while C(ext) corresponds to the peripheral carbon atoms of the cumulene ligand. Orbitals A (J), B (K), and C(L) are the bonding (anti-bonding) orbitals built from the π bond in the cumulene ligand. Orbitals D-I are metal-based orbitals, with dominant f character.

Supplementary Table 12. Electronic wave function of the ground spin-orbit state, in terms of determinants for the Np derivative. Only determinants contributing by more than 1% are shown. The spin component is neglected. Determinants are written down with respect to the occupation number of the different f orbitals shown in Supplementary Figure 33.

orbitals/ weight	A	B	C	D	E	F	G	H	I	J	K	L
38%	2	2	2	1	1	1	0	0	0	0	0	0
25%	2	2	2	1	0	1	1	0	0	0	0	0
6%	2	2	2	0	1	1	0	1	0	0	0	0
6%	2	2	2	0	1	1	0	0	1	0	0	0
4%	2	2	2	0	0	1	1	0	1	0	0	0
2%	2	2	2	1	1	0	0	1	0	0	0	0
2%	2	2	2	1	0	0	1	1	0	0	0	0

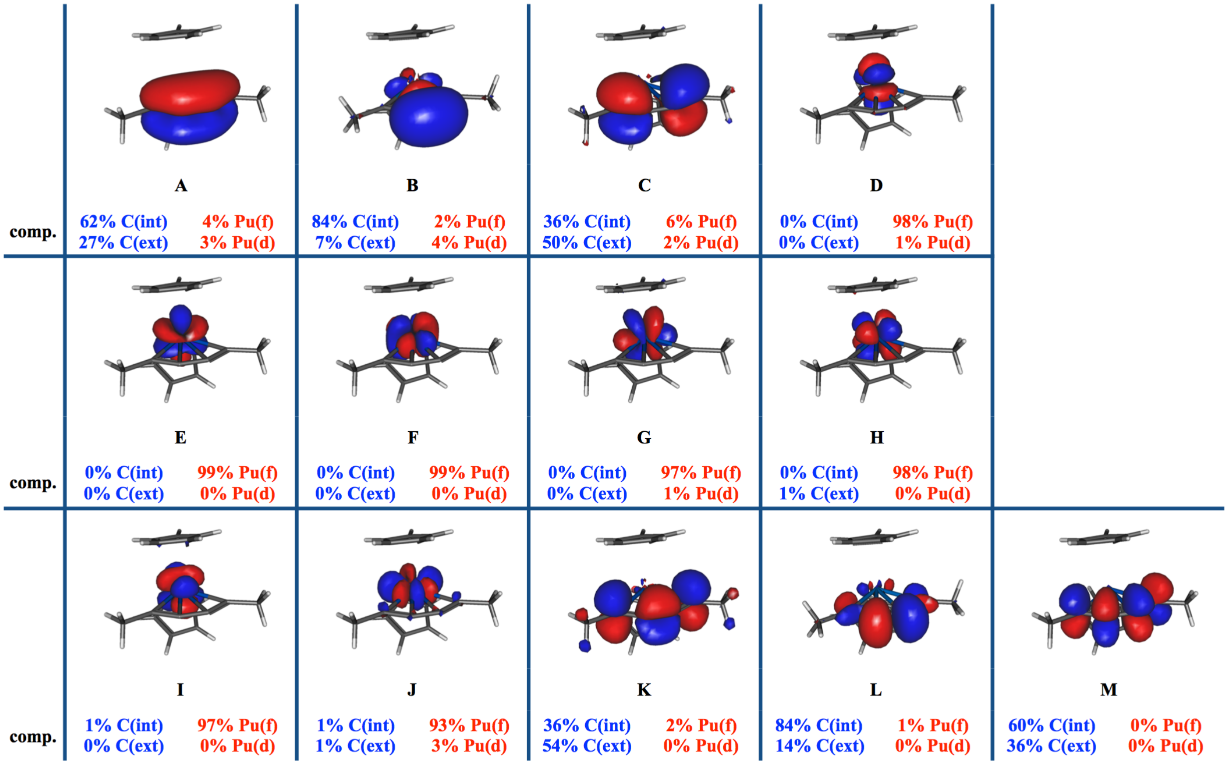

Supplementary Figure 34. Active space MOs for the Pu derivative, with their composition in terms of atomic orbitals (isosurface = 0.03). C(int) corresponds to the internal carbon atoms, while C(ext) corresponds to the peripheral carbon atoms of the cumulene ligand. Orbitals A (K), B (L), and C(M) are the bonding (anti-bonding) orbitals built from the π bond in the cumulene ligand. Orbitals D-J are metal-based orbitals, with dominant f character.

Supplementary Table 13. Electronic wave function of the ground spin-orbit state, in terms of determinants for the Pu derivative. Only determinants contributing by more than 1% are shown. The spin component is neglected. Determinants are written down with respect to the occupation number of the different f orbitals shown in Supplementary Figure 34.

orbitals/ weight	A	B	C	D	E	F	G	H	I	J	K	L	M
40%	2	2	2	1	1	1	0	1	0	0	0	0	0
29%	2	2	2	1	1	1	1	0	0	0	0	0	0
6%	2	2	2	0	1	1	0	1	1	0	0	0	0
3%	2	2	2	0	1	1	1	0	1	0	0	0	0
3%	2	2	2	1	0	0	1	1	0	1	0	0	0
2%	2	2	2	1	0	1	0	1	1	0	0	0	0
2%	2	2	2	1	0	1	1	0	1	0	0	0	0
2%	2	2	2	1	0	1	0	1	0	1	0	0	0
1%	2	2	2	0	0	1	1	0	1	1	0	0	0

For all systems, the electronic wave function of the ground state has a strong multi-reference character, which stems from distributing the unpaired electrons among the orbitals with dominant f-character, but does not involve the ligand orbitals. Indeed, the occupation number of the ligand-based orbitals in any determinant that makes a contribution larger than 1% is always either 2 or 0, which means that these orbitals do not contribute to the multi-reference character of the wave function.

In addition, from the detailed composition of the active space orbitals, it appears that orbitals with dominant f-character are mostly non-bonding as they always hold close to 99% f character. The only noticeable exception is the Pa derivative for which bonding-type metal-ligand mixing (96% vs. 3%) is observed, as shown on Supplementary Figure 35-a. The f orbital that is involved in this mixing has the same symmetry as the one evidenced from DFT calculations and AdNDP analysis to achieve φ back-donation.

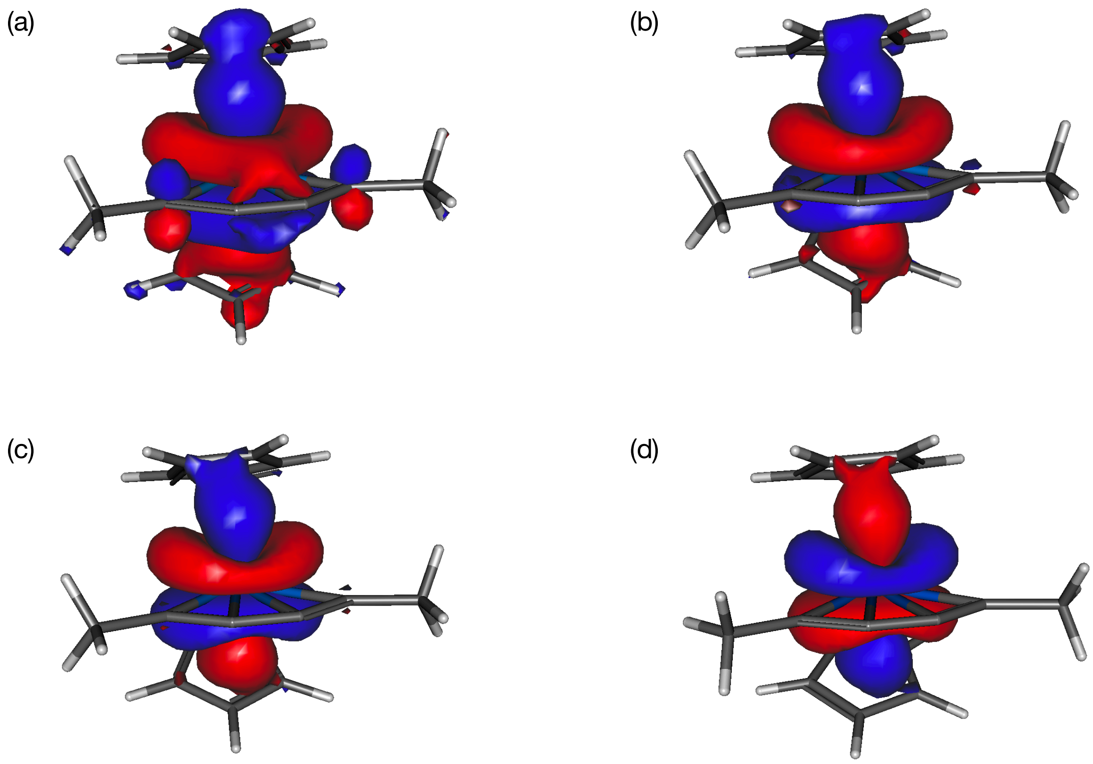

Supplementary Figure 35. Orbital F of the Pa derivative (a), and orbital E of the U, Np and Pu derivatives (b-d) with isosurface = 0.01.

Since (1) the metal-based orbitals of the active space are mostly non-bonding, and (2) the multi-reference character of the ground state wave function stems from distributing the unpaired electrons among these non-bonding orbitals, it is expected that the multi-reference character of the wave function will not modify the bonding picture obtained from the AdNDP analysis based on single-reference DFT calculations for the investigated systems.

Supplementary References

(1) Zhang, L.; Fang, B.; Hou, G.; Zi, G.; Ding, W.; Walter, M. D. *Organometallics* 2017, *36*, 898–910.

(2) Pagano, J. K.; Erickson, K. A.; Scott, B. L.; Morris, D. E.; Waterman, R.; Kiplinger, J. L. *J. Organomet. Chem.* 2017, *829*, 79–84.

(3) Pellny, P. M.; Kirchbauer, F. G.; Burlakov, V. V.; Baumann, W.; Spannenberg, A.; Rosenthal, U. *J. Am. Chem. Soc.* 1999, *121*, 8313–8323.

(4) Fang, B.; Zhang, L.; Hou, G.; Zi, G.; Fang, D. C.; Walter, M. D. *Organometallics* 2015, *34*, 5669–5681.

(5) Burlakov, V. V.; Polyakov, A. V.; Yanovsky, A. I.; Struchkov, Y. T.; Shur, V. B.; Vol'pin, M. E.; Rosenthal, U.; Görls, H. *J. Organomet. Chem.* 1994, *476*, 197–206.

(6) Fang, B.; Ren, W.; Hou, G.; Zi, G.; Fang, D. C.; Maron, L.; Walter, M. D. *J. Am. Chem. Soc.* 2014, *136*, 17249–17261.

(7) Zhang, L.; Hou, G.; Zi, G.; Ding, W.; Walter, M. D. *J. Am. Chem. Soc.* 2016, *138*, 5130–5142.

(8) Arndt, P.; Burlakov, V. V.; Spannenberg, A.; Rosenthal, U. *Inorg. Chem. Commun.* 2007, *10*, 792–794.
